# Selective consumption of sacoglossan sea slugs (Mollusca: Gastropoda) by scleractinian corals (Cnidaria: Anthozoa)

**DOI:** 10.1371/journal.pone.0215063

**Published:** 2019-04-29

**Authors:** Rahul Mehrotra, Coline Monchanin, Chad M. Scott, Niphon Phongsuwan, Manuel Caballer Gutierrez, Suchana Chavanich, Bert W. Hoeksema

**Affiliations:** 1 Reef Biology Research Group, Department of Marine Science, Faculty of Science, Chulalongkorn University, Bangkok, Thailand; 2 New Heaven Reef Conservation Program, Koh Tao, Suratthani, Thailand; 3 Department of Marine and Coastal Resources, Bangkok, Thailand; 4 Muséum National d’Histoire Naturelle, Directions des Collections, Paris, France; 5 American University of Paris, Department of Computer Science Math and Environmental Science, Paris, France; 6 Center for Marine Biotechnology, Department of Marine Science, Faculty of Science, Chulalongkorn University, Bangkok, Thailand; 7 Taxonomy and Systematics Group, Naturalis Biodiversity Center, RA Leiden, The Netherlands; Biodiversity Research Center, TAIWAN

## Abstract

Recent studies revealed that reef corals can eat large-sized pelagic and benthic animals in addition to small planktonic prey. As follow-up, we document natural ingestion of sea slugs by corals and investigate the role of sacoglossan sea slugs as possible prey items of scleractinian corals. Feeding trials were carried out using six sacoglossan species as prey, two each from the genera *Costasiella*, *Elysia* and *Plakobranchus*, and four free-living solitary corals (*Danafungia scruposa*, *Fungia fungites*, *Pleuractis paumotensis* and *Heteropsammia cochlea*) as predators. Trials were carried out under both in-situ and ex-situ conditions with the aim to observe ingestion and assess signs of prey consumption based on tissue loss of prey individuals over time. Significant differences were observed in both ingestion time and consumption state of prey between prey species, with three of them being ingested more rapidly and preferentially consumed over the others. Additionally, prey size was found to be a significant factor with larger prey (>12 mm) being ingested more slowly and rarely than smaller ones (<6 mm and 6–12 mm). Comparisons of consumption capability among predators showed no significant difference with all coral species showing similar preferences for prey species. While no specific mechanism of prey capture is proposed, we also document instances of kleptoparisitism and resuspension of prey items by wrasses. This study highlights the important distinction between opportunistic prey capture and true predation events.

## Introduction

Sea slugs (Mollusca: Gastropoda: Heterobranchia) are more commonly known as predator than as prey, which is largely attributed to the chemical defences acquired from their prey species [1–3]. Though less extensive, a growing number of instances of sea slugs as prey species have been recorded, as part of in-situ observations and experiments, in particular with cnidarian predators [4,5]. However, once a prey is captured, it is not sure that it will also be consumed because it may become released and eventually escape [6].

Sacoglossans are a speciose clade of sea slugs that feed almost exclusively on algal matter [7]. A small number of sacoglossans species have been found to be predated upon by a variety of organisms including small fish, nemerteans, crustaceans, a scleractinian coral, and other sea slugs [5, 8–10]. These predatory organisms either display a high degree of tolerance to sequestered toxins in prey species or are completely unaffected by them, with a few cases known of prey rejection due to sacoglossan toxins [2,8].

Heterotrophy in scleractinian reef corals plays a vital role in the growth of coral tissue, even though this usually plays an inferior role compared to energy acquisition through autotrophy [11,12]. Heterotrophy can meet 15–35% of daily metabolic requirements in healthy zooxanthellate corals and up to 100% in bleached corals, and can account up to 66% of carbon used in their skeletons [12]. Key nutritional importance of heterotrophy has been reported to include acquisition of amino acids and nutrients such as nitrogen and phosphorus as these are not acquired by the coral via photosynthesis. Prey composition of scleractinian predators is composed largely of zooplankton and particulate organic matter (POM) of various sizes. Zooplankton prey include isopods, amphipods, crab larvae, copepods, nematodes, nemerteans, polychaetes, and jellyfish [13–17].

While the mechanics of prey digestion have received some attention, prey rejection in scleractinian corals has not been studied in great detail. Studies into predation and dietary preferences have been conducted for well over a century [18–20] with some sparse mention of prey rejection. Notably de Lecaze-Duthiers [18] documented ingestion and subsequent rejection of live whelks, *Nucella lapillus*, during feeding trials on the dendrophylliid coral *Balanophyllia regia*. Subsequent observations made on the subject include notes of ‘gut content discharge’ [14] and the rejection of prey under varying conditions/stimuli [21–23].

In recent years, a growing number of observations have shown how large planktonic fauna such as salps and jellyfish may act as prey for corals [24–28]. These records suggest that gape size in corals directly limits the range of potential prey items [26]. Nearly all of the predatory corals observed so far, appear to be free-living, allowing them to live in various reef zones, including deeper sandy seafloors [29–31]. These free-living corals are mobile and can also shed sediments relative easily [32–34].The ability to survive on unconsolidated substrates enables free-living corals to expand the reef habitat by means of spreading their skeletons as hard pieces of substrate in downslope direction beyond the lower reef slope boundary in an ecological and evolutionary context [30,35,36]. A number of free-living zooxanthellate scleractinian corals of various families are known to live almost exclusively in soft sediment non-reef habitats, such as *Heteropsammia* spp. (Dendrophylliidae), *Heterocyathus* spp. (Caryophylliidae), *Cycloseris* spp. (Fungiidae), and *Goniopora stokesi* (Poritidae) [30, 36–38]. There are also some free-living coral species without zooxanthellae that can thrive on sandy bottoms near reefs but usually live in much deeper and colder water, such as *Truncatoflabellum* spp. (Flabellidae) and *Deltocyathoides* spp. (Turbinoliidae) [39–41]. Little is known about the ecology of these low-cover, soft-bottom communities near reefs, which have been considered to play a limited role in reef-building [35]. It remains unclear whether the ability to survive in these habitats for these corals is linked to their gape size and thus, to their predatory capacity of larger prey items.

Free-living corals of the species *Heteropsammia cochlea* and *Pleuractis paumotensis* were recently observed to ingest salps and a sea slug at Koh Tao, Gulf of Thailand [5,26]. The 19-km^2^ island is surrounded by a fringing coral reef, with many of the locations around the island supporting large populations of free-living mushroom corals [41–43]. Beyond the reef slope there is a soft-sediment ecosystem that supports mixed aggregations of free-living scleractinian corals such as *Heteropsammia cochlea* and *Heterocyathus aequicostatus* [26,31]. This ecosystem has been shown to support a hidden diversity of sea slugs and other fauna that are not present within the shallower reef habitats, with over a third of all species of sea slug recorded from the island exclusively recorded from this ecosystem [44, 45]. To date, seven species of sacoglossan sea slug are known from Koh Tao [44], including two species of *Plakobranchus*. The recent description of *Plakobranchus papua* [45] has helped identify a third cryptic species living in sympatry with *Plakobranchus* cf. *ocellatus* at Koh Tao, herein referred to as *Plakobranchus* cf. *papua*. Additionally, two species of the sacoglossan sea slug genus *Costasiella* (*C*. *usagi* and *C*. cf. *kuroshimae*) and two species of *Elysia* (*E*. *asbecki* and *E*. cf. *japonica*) were recorded.

Based on the previous information [5,12,24–27], it is clear that the role of large-mouthed corals as consumers, or at least as opportunistic predators, does not follow conventional understanding and requires further investigation. Moreover, with even fewer records existing of sacoglossan sea slugs as prey species, confirmed records of scleractinian coral consumption of sea slug taxa would place a previously unknown ecological role upon sea slugs in the food cycle of coral reef ecosystems. Herein we explore the potential of such a relationship by means of three objectives. Firstly, by use of both in-situ and ex-situ trials, we aim to ascertain the palatability of various sacoglossan species by large-mouthed solitary corals, which will be done by conducting feeding trials and measuring responses to prey items. Secondly, we aim to investigate the role of habitat type on such relationships by utilising both predators and prey from two contrasting ecosystems, namely coral reefs and deeper soft-sediment habitats [5,26,44]. Thirdly, we aim to assess the method and rate by which prey captured by large monostomatous (solitary) mushroom corals are transported from point of capture to the mouth.

## Methodology

### Sampling and prey selection

**Fig 1:**
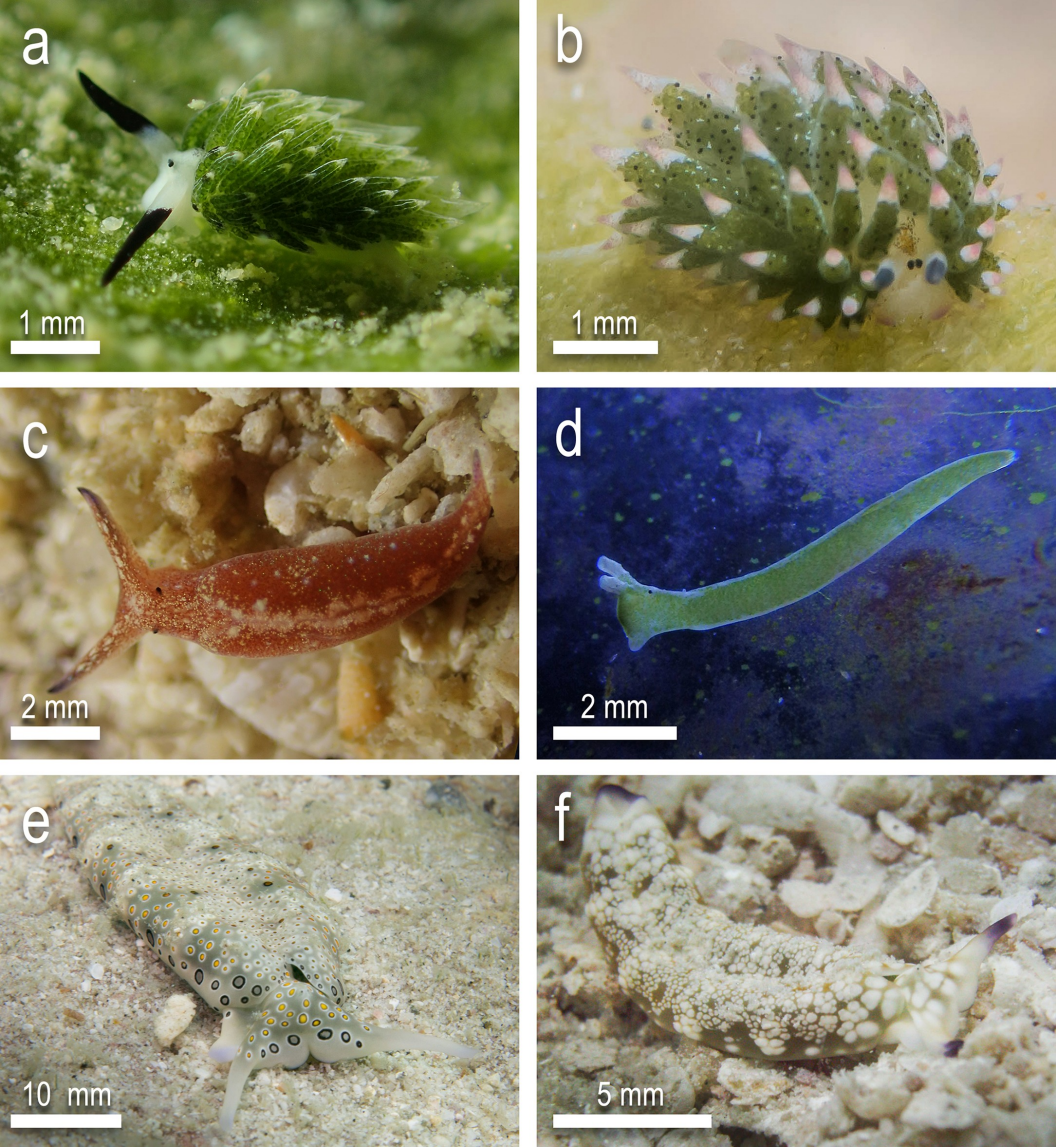
Prey species tested for consumption by corals. a) *Costasiella usagi* b) *Costasiella* cf. *kuroshimae* c) *Elysia* cf. *japonica* d) *Elysia pusilla* e) *Plakobranchus* cf. *ocellatus* f) *Plakobranchus* cf. *papua*.

**Fig 2:**
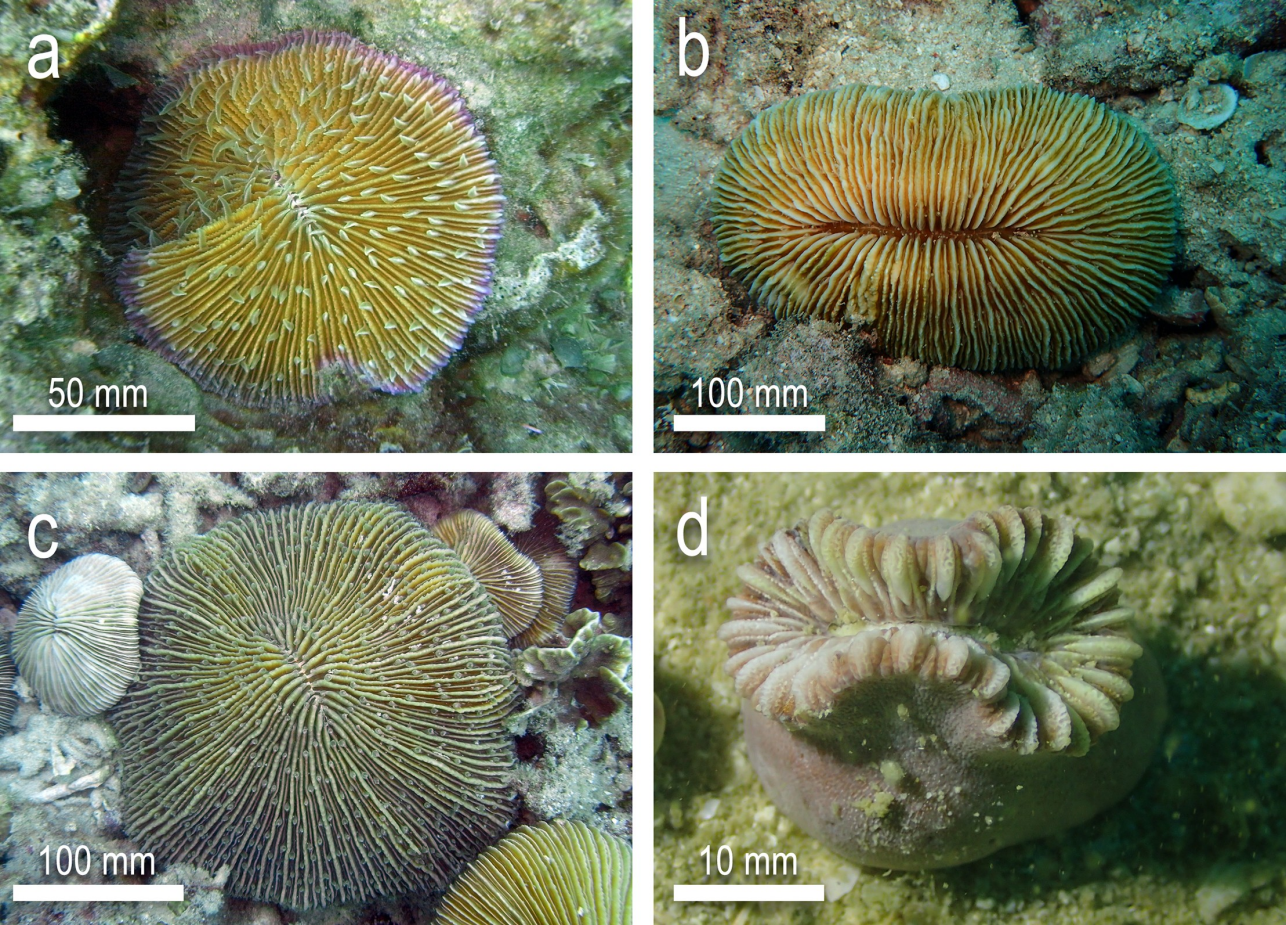
Predator corals tested for consumption of slugs. a) *Fungia fungites* b) *Pleuractis paumotensis* c) *Danafungia scruposa* d) *Heteropsammia cochlea*.

To evaluate whether sea slugs have a trophic role in the diet of solitary scleractinian corals, feeding trials were carried between numerous species of coral and sea slug. Sampling was carried out by hand using SCUBA and snorkelling with all sampling and feeding trials conducted from April to August between 2015 and 2017. Specimens belonging to six species of sacoglossan sea slugs were used in the trials. These prey species were: *Elysia pusilla*, *Elysia* cf. *japonica*, *Costasiella* cf. *kuroshimae*, *Costasiella usagi*, *Plakobranchus* cf. *papua* and *Plakobranchus* cf. *ocellatus* (Fig 1). All were collected from their natural habitat (Table 1) and, if needed, kept in a holding tank for a short period of time before the trials. Three of the sacoglossans are found exclusively in the deeper soft sediment habitats (depth from 10 m to >16 m) of Koh Tao (*C*. cf. *kuroshimae*, *C*. *usagi* and *E*. cf. *japonica*) with the other three species found exclusively at the reef edge and on shallow soft sediment habitats (0.5 m to 10 m). These species were selected because they represent the most abundant sacoglossan taxa at Koh Tao while also representing variation in life history and ecology [44]. For the coral predators, 60 specimens of each of the four free-living coral species chosen for ex-situ trials were collected during the same sampling period as the sea slugs. Free-living monostomatous mushroom corals (*Fungia fungites*, *Danafungia scruposa*, and *Pleuractis paumotensis*) were sampled at depths corresponding to their greatest abundance, at 3–8 m, while *Heteropsammia cochlea* individuals were sampled likewise at depths of greatest abundance, at 12–16 m (Fig 2). Identifications were based on numerous recent faunistic investigations [44,46–50]. All corals were sampled at random using roving diver surveys [51] with the only prerequisite criteria being the given coral species and health of coral (absence of disease, recent physical trauma or bleaching).

**Table 1:** Sampling sites at Koh Tao, Gulf of Thailand, indicating which predator and prey species were sampled from which location and total depth range of sampling. In-situ trials were conducted at all sites.

Location	Latitude	Longitude	Prey Species	Predator Species	Depth Range (m)
Tien Og Bay	10°03′46″N	99°50′01″E	*Plakobranchus* spp.	Fungiidae spp.	3–8
Chalok Bay	10°03′41″N	99°49′30″E	*Elysia pusilla* and *Plakobranchus* spp.	Fungiidae spp.	3–8
Tao Tong	10°03'54"N	99°49'14"E	*Plakobranchus* spp., *Costasiella* spp. and *Elysia japonica*	Fungiidae spp.	3–16
Aow Leuk	10°04′11″N	99°50′32″E	*E*. *japonica* and *Costasiella* spp.	*Heteropsammia cochlea*	10–16
Sai Nuan Bay	10°04′38″N	99°48′47″E	*E*. *japonica* and *Costasiella* spp.	All species	3–16

Corals were held in aquarium tanks (approx. 20,000 cm^3^) with sea water changed every day and with temperature and salinity monitored every 6–8 hours (ranging from 29–31°C and 34.5–36.5‰ throughout the experiment). Individuals of *H*. *cochlea* were kept in aquaria composed of a sand and silt substrate collected from their original habitat. Holding tanks for sacoglossans (volume 800 cm^3^, surface area approx. 500 cm^2^) contained new sea water (also temperature-monitored, changed daily) and species-specific algal food when possible. Individuals were held, when needed, at low densities (maximum density of three individuals for *Plakobranchus* spp. and four individuals each for *Costasiella* spp. and *Elysia* spp., with minimal overlap of species being held. Sacoglossan prey sizes were measured based on maximum length from anterior extent to posterior extent of the foot and were measured during locomotion prior to collection. Measurements were conducted again immediately before trial to account for any change in size. Sizes were divided into three classes based on maximum length, regardless of species: Class 1 contained the smallest individuals (< 6 mm), Class 2 contained larger individuals (6 to 12 mm), and Class 3 contained the largest individuals, 12 to 45 mm.

### Ethics statement

All surveys and experiments carried out full comply with the regulations and ethical guidelines of Chulalongkorn University, Bangkok, Thailand, with all necessary permissions acquired and no guidelines breached. Site specific permissions were given by the Department of Marine and Coastal Resources and the Department of Fisheries of Thailand.

### Feeding trials

To evaluate whether sea slugs have a trophic role in the diet of solitary scleractinian corals, in-situ feeding trials were conducted in-situ via SCUBA at depths of 3–8 m for mushroom corals and 10–16 m for *H*. *cochlea*. In addition, trials were conducted ex-situ with the four coral species in tanks to increase duration of observation. To assess the priority of factors playing a role in prey selection, trials were conducted based on the separation of two primary variables, prey species and prey size. In addition, the three mushroom coral species in the trials were used to identify any potential differences in prey selection among coral species. Each species was tried an equal number of times but without any bias in prey species or size. The soft-sediment species *Heteropsammia cochlea* was used in both in-situ and ex-situ trials. For in-situ trials, 10 replications per prey species were used, and another 10 were used in the ex-situ trials. In total, 40 individuals of each prey species were included in the experiments (20 each for the fungiid species and *H*. *cochlea*, with 10 tried in-situ and 10 ex-situ for each). Predator corals were randomly distributed among prey items in order to avoid inherent bias between prey size or between predator species, such that all predator corals were tried an equal number of times and all prey items were tested an equal number of times as well (S1–S4 Tables).

**Fig 3:**
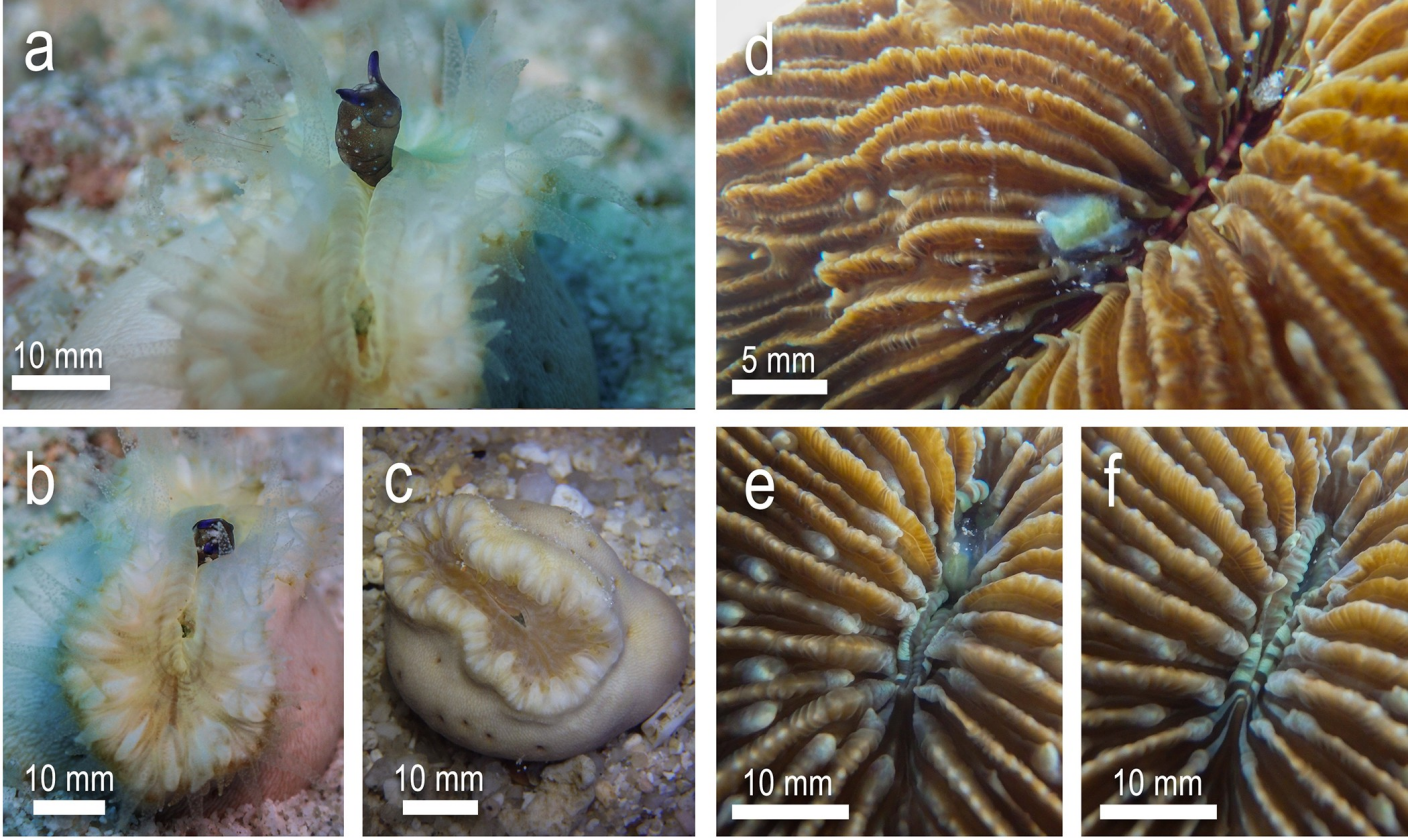
**Ingestion of sacoglossan species *Elysia* cf. *japonica* by *Heteropsammia cochlea*** (a-c) **and *Elysia pusilla* by *Fungia fungites*** (d–f).

Each prey individual was tried with a different coral and was observed as long as possible within the constraints of SCUBA limits, or until a given prey was rejected by the coral. Single prey individuals were placed on, or in the vicinity of the mouth of the coral and were timed until ingestion was complete (Fig 3). Completed ingestion was defined as when a prey item was inside a closed coral mouth with less than approximately 5% of the prey item remaining visible when observed from above. Completed rejection was defined as when a prey item was more than 95% visible and above the mouth of the coral, with the mouth closed or closing beneath the prey item such that less than approximately 5% of the prey remained within the mouth.

In-situ trials were conducted during the sampling period and were carried out at day time, between 10:00 and 15:30, on healthy corals ready to feed, which was determined visually by the presence of expanded tentacles and an open mouth. Ex-situ trials were conducted in separate tanks (20,000 cm^3^) between 12:00 and 24:00, when corals were visually assessed as ready to feed. Pilot studies indicated no variation in measurements based on time of feeding and as such was omitted as a variable during the study. It should be noted that in-situ feeding trials were attempted at night repeatedly. The use of artificial light for observation contributed heavily to the density of fauna attracted to the light, and therefore any given coral used in a feeding trial was rapidly swarmed. Corals were then found to actively ingest the numerous zooplankton, worms and other fauna caught in its mouth and tentacles, making visual monitoring of any particular prey item nearly impossible. Ex-situ trials were conducted in aquaria when coral tentacles were extended and coral mouths open. Trials were carried out to best mimic in-situ conditions and all trials were observed by eye to estimate a change in the state of predation. Temperatures in aquaria mimicked environmental conditions (29–31 ^o^C), as is typical for the months during which in-situ trials were conducted.

**Fig 4:**
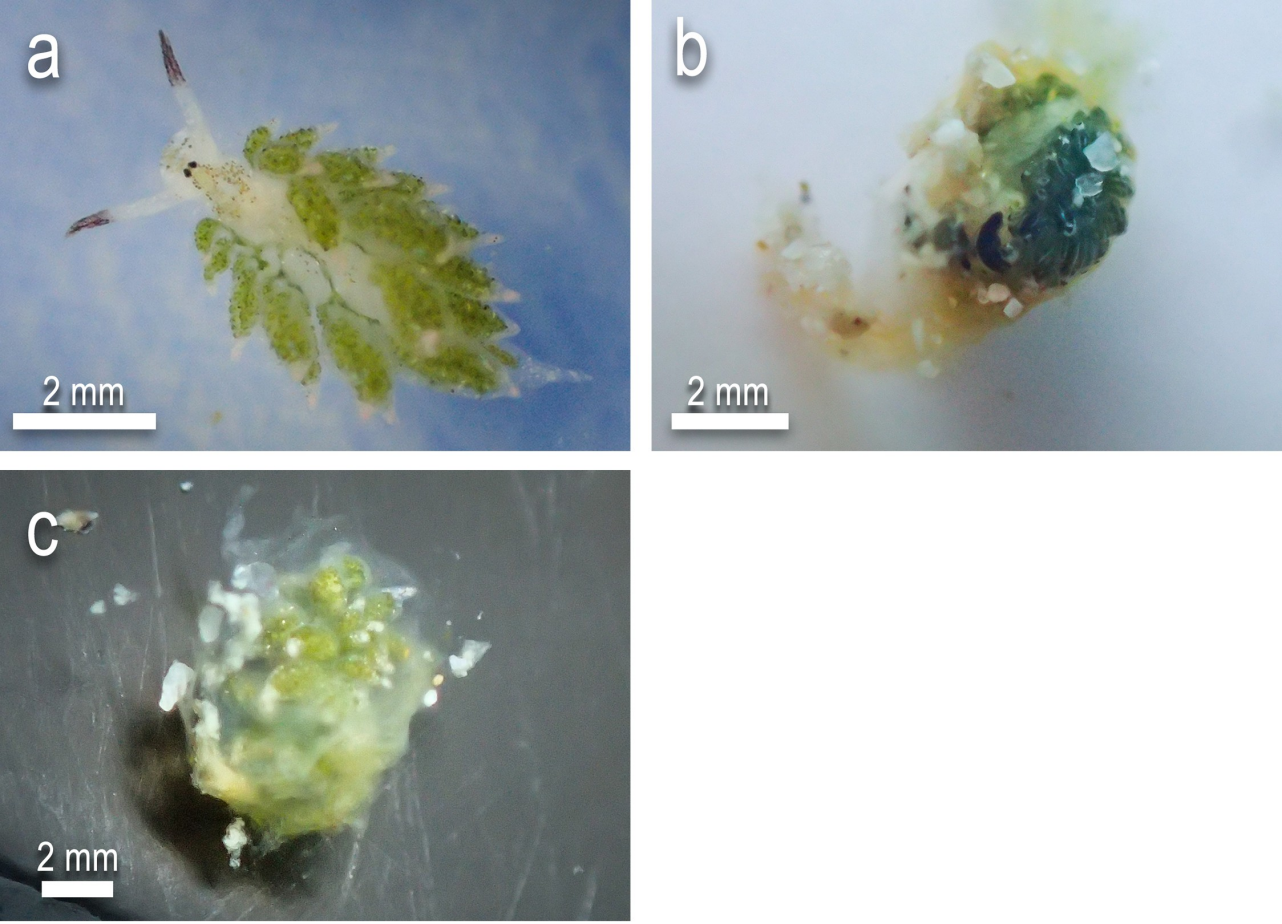
States of consumption of prey items upon extraction from coral mouth. a) Consumption score 0 showing minimal signs of tissue degradation b) Consumption score 0.5 indicating partial tissue breakdown and c) Consumption score 1 signifying heavy tissue loss.

Size data were collected on the maximum length of predator coral, prey slug and maximum size of gape, which was determined to be the greatest length of tissue that can be opened for the purpose of prey ingestion. Time data was taken from the point of contact between the mouth of predator and prey item. Time until complete ingestion or complete rejection were also taken, alongside post-ingestion observation length (PIOL), which was required due to the limitations in observation length based on SCUBA constraints. After a PIOL was determined for non-rejected slugs, prey was extracted (where possible) from inside the coral mouth, to assess the degree of digestion, referred to as the consumption score (Fig 4). Extracted prey were given a score of 0 for minimal signs of tissue loss, 0.5 for partial digestion as estimated by eye, and a score of 1 for prey with high degree of tissue degradation, leaving the prey unrecognisable (Fig 4). Rejected prey were assessed under the same scoring.

### Prey transport

**Fig 5:**
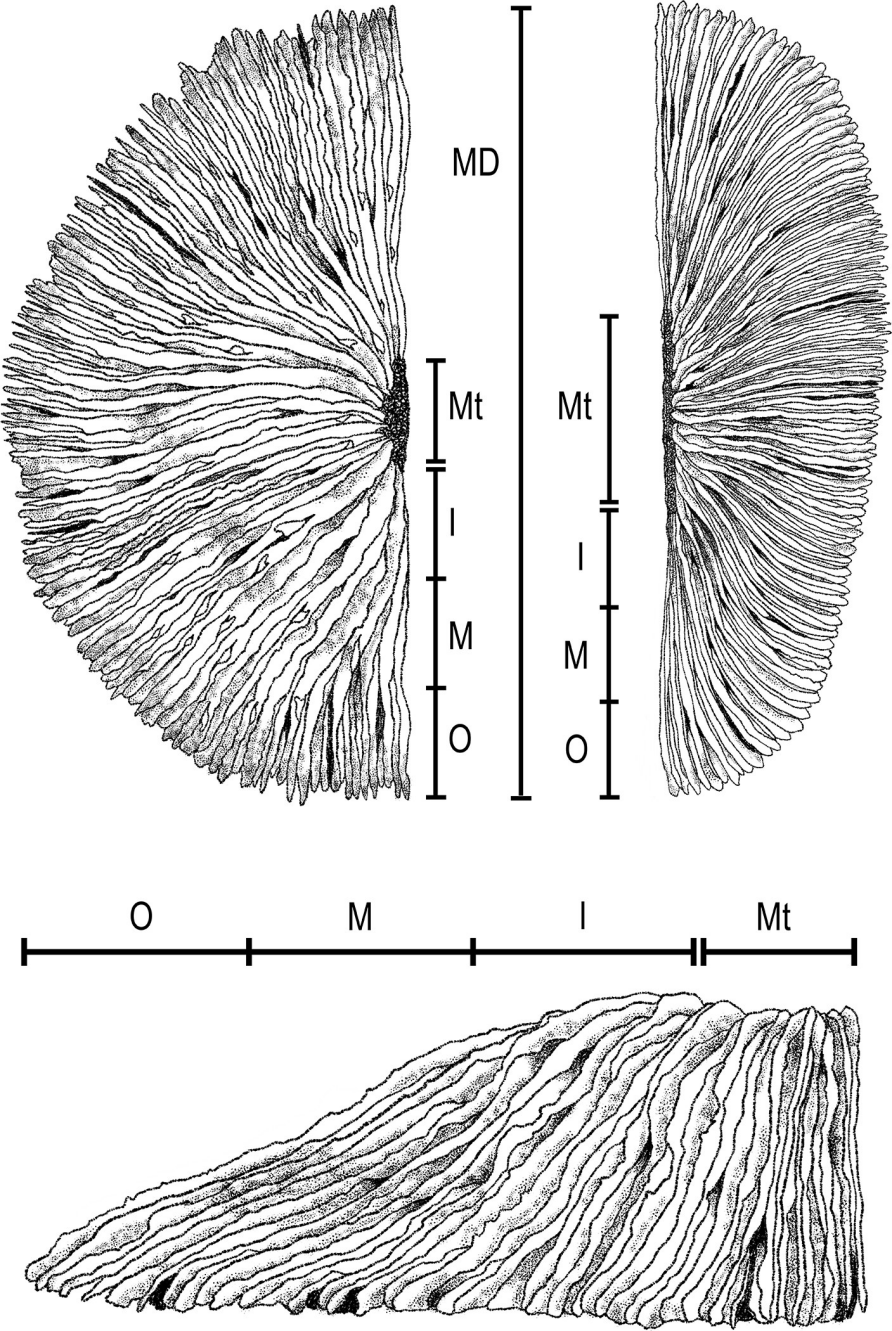
**Measurements taken of Fungiid corals *Fungia fungites* (top left) and *Pleuractis paumotensis* (top right) for the purpose of assessing rates of prey transport.** Mt–Mouth, I–Inner Coral, M–Middle Coral, O–Outer Coral, MD–Maximum Diameter.

Another study was carried out to observe the means by which prey was transported from different parts of the oral surface of different mushroom corals towards the mouth [52,53]. The rate of movement across the coral was calculated as mm/min. Prey items were placed on the outer, middle or inner parts of the mushroom coral’s oral side, in line with the axis of the mouth to maximise relative distance observed (Fig 5). These were calculated as follows:

(Max Diameter of Coral–Mouth Length)/2 = Max. Distance from perimeter of coral to mouth.

This maximum distance from perimeter was assumed to be the greatest linear distance a prey item would have to travel once captured by a coral, to the mouth [53]. Dividing this distance into three equal distances allowed for categorising ‘Outer’, ‘Middle’ and ‘Inner’ regions. The maximum diameter of the coral always took into account slope, for instances where the coral was particularly convex, and always the greatest distances across oval corals such as *P*. *paumotensis* (Fig 2B).

The time from point of capture by the coral, to the first contact with the mouth was measured. Comparisons of rates of transport between the different prey species and different predator coral were made in both in-situ and ex-situ trials (27 and 10 trials, respectively). Prey transport trials were independent of feeding trials so as to not alter physical state of a prey item, which may influence perceived consumption state of prey after ingestion, thus number of prey transport trials were limited by the number of separate prey items available for assessment. Given the dependence on wave motion in prey transport during in-situ experiments (see discussion), wave action was mimicked manually from the point of introduction of a prey item until a given prey item was successfully transported to the mouth during ex-situ experiments. Variation in rate of movement by prey species over coral surface were analysed. Prey transport experiments were not carried out on *H*. *cochlea* corals, as the majority of dorsal surface of these corals is made up of a large mouth lined with a thin row of long tentacles, therefore leaving little space for any particular prey item to be transported along.

### Statistical tests

Two-way ANOVA tests were carried out to assess significance alongside Kruskal Wallis tests on non-parametric data, with a post hoc comparison using the Wilcoxon-Mann-Whitney test. Differences with p < 0.05 were considered statistically significant. Pearson’s tests were used to assess correlation and r > 0.5 were considered statistically significant.

## Results

**Fig 6:**
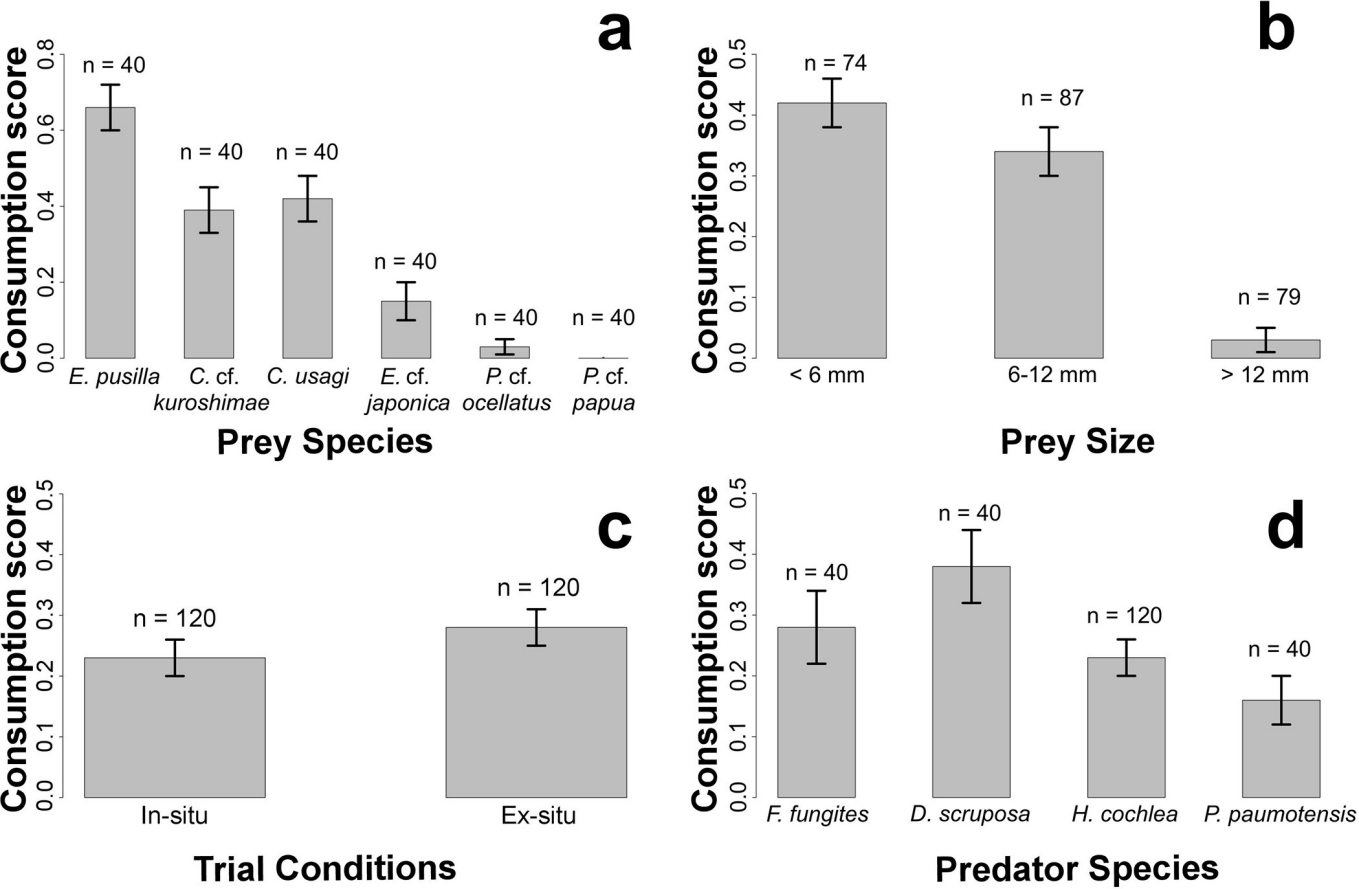
**Comparison of mean consumption scores between a) prey sacoglossan species b) prey size classes c) in-situ and ex-situ feeding trials and d) predator corals.** Error bars represent standard error and y-axis indicates consumption score from 0 (no visible tissue loss) to 1 (heavy tissue degradation).

Among the six sacoglossan prey species, three were found to be consumed more frequently than rejected (Table 2): *Elysia pusilla*, *C*. *usagi* and *C*.cf. *kuroshimae* were only rejected in 2.5%, 20% and 17.5% of trials, respectively. Additional trials of over 120 mins were carried out on each to confirm that these species were not being rejected at a later point. Under in-situ conditions, the mean PIOL for all non-rejected trials was 62 mins and under ex-situ conditions, the mean PIOL for all non-rejected trials was 131 mins. Mean consumption score for *E*. *pusilla*, *C*. *usagi* and *C*.cf. *kuroshimae* (Fig 6) independent of coral species, indicates that after an hour, individuals typically showed at least some tissue degradation, with *E*. *pusilla* showing significantly greater consumption scores than all other prey species. In contrast, *E*. cf. *japonica* rarely showed signs of consumption, being rejected 52.5% of the time with a mean consumption score of 0.15 ± 0.05. Both *Plakobranchus* species were typically rejected, with *P*. cf. *ocellatus* and *P*. cf. *papua* being rejected 95% and 90% and having consumption scores of 0.03 ± 0.02 and 0 respectively. These scores remain low, despite sparse instances where prey items were not rejected, as upon extraction prey items showed little to no signs of tissue degradation, and multiple instances where ingestion remained incomplete. With all trials for all prey species combined, most rejected prey were found to have a consumption score of 0 (n = 77), with the remaining having a consumption score of 0.5 (n = 6) and all rejected prey items were alive post rejection and motile shortly after rejection. No rejected prey items showed heavy signs of tissue degradation.

**Table 2:** On the left, mean duration of ingestion and mean time to rejection per species in minutes and proportion of prey items rejected (including incomplete ingestion) as % of total trials per species. On the right, pairwise Wilcoxon rank sum *p* values for comparison between prey species for the consumption scores. Significant results were considered as those with *p* < 0.05.

Mean Ingestion Duration (min. ± SE)	Mean Rejection Time (min. ± SE) and %	Prey Species	*C*. *usagi*	*C*. cf. *kuroshimae*	*E*. cf. *japonica*	*E*. *pusilla*	*P*. cf. *ocellatus*
3.03 ± 0.31	27.63 ± 2.29 (20%)	*C*. *usagi*	**-**	-	-	-	-
4.28 ± 0.72	13 ± 0.75 (17.5%)	*C*. cf. *kuroshimae*	0.7048	-	-	-	-
3.3 ± 0.62	20.95 ± 2.18 (52.5%)	*E*. cf. *japonica*	**< 0.01**	**< 0.05**	-	-	-
7.1 ± 1.07	18 ± 0.45 (2.5%)	*E*. *pusilla*	**< 0.05**	**< 0.05**	**< 0.001**	-	-
17.06 ± 1.65	28.64 ± 2.60 (95%)	*P*. cf. *ocellatus*	**< 0.001**	**< 0.001**	0.0849	**< 0.001**	-
6.9 ± 0.69	14 ± 1.35 (90%)	*P*. cf. *papua*	**< 0.001**	**< 0.001**	**< 0.05**	**< 0.001**	0.3198

Based on the availability of prey sizes, the number of individuals of size class 1, 2 and 3 were 74, 87 and 79 respectively (Fig 6B). No significant difference was recorded for consumption values between prey classes 1 and 2 (p = 0.36) which included multiple representatives of all species. However, both were significantly different from degree of consumption observed in size class 3, those individuals greater than 12 mm to the largest size of 45 mm (p values for pairwise comparison between class 1 and 3, and 2 and 3 are < 0.01 in both instances). These largely included both *Plakobranchus* species alongside numerous individuals from both *Elysia* species. Similarly, while no significant differences were attributed to ingestion time between classes 1 and 2, both were found to be significantly different with the largest prey items with size class 1 taking approximately 3.7 minutes on average (± 0.5 min) to complete ingestion, class 2 taking approximately 5.8 min (± 0.6 mins) and class 3 taking on average greater than 10.1 min (± 0.9 min) to complete ingestion (Table 3). Pairwise comparisons of ingestion time between class 1 and 2 resulted in p = 0.4, between 1 and 3 in p < 0.001, and between 2 and 3 in p < 0.01.

**Table 3:** Comparison of ingestion duration and time to rejection between three size classes of prey, four species of coral predator and between in-situ and ex-situ feeding trials.

Prey size class	Mean Ingestion Duration ± SE (min)	Mean Rejection Time ± SE (min)
1: < 6 mm	3.72 ± 0.47	21.53 ± 1.34
2: 6–12 mm	5.80 ± 0.63	21.04 ± 1.19
3: >12 mm	10.05 ± 0.89	20.84 ± 1.54
**Predator Coral**		
*Danafungia scruposa*	5.39 ± 1.17	28.57 ± 2.46
*Fungia fungites*	7.79 ± 1.28	20.63 ± 1.89
*Pleuractis paumotensis*	5.97 ± 0.73	20.26 ± 1.87
*Heteropsammia cochlea*	5.03 ± 0.47	18.45 ± 1.04
**Trial type**		
In-Situ	6.25 ± 0.61	18.86 ± 1.03
Ex-Situ	5.34 ± 0.51	23.41 ± 1.24

With regards to predatory capacity of coral species, mean consumption scores ranged from 0.16 ± 0.04 to 0.38 ± 0.06 and no significant difference was found between them (Fig 6C). Among the fungiid predators, no significant differences were observed between species with regards to consumption scores, nor mean ingestion durations which ranged from 5.39 ± 1.2 min in *D*. *scruposa* to 7.8 ± 1.3 min in *F*. *fungites*. Additionally, no coral was found to take significantly more time to reject prey items than the any other. When comparing in-situ and ex-situ trials, Kruskal-Wallis tests revealed no significant differences in consumption scores (p = 0.47), ingestion times (p = 0.30) and rejection times (p = 0.17).

Significant differences were found in the mean consumption scores and ingestion duration between the slug species and the separate prey size classes, and time to rejection was significantly different among the prey species but not between the size classes as inferred by the Kruskal Wallis test and Wilcoxon-Mann-Whitney test (Table 4). Differences in length of time until rejection were also found to be significant between *Heteropsammia cochlea* trials and the combined data of the three Fungiidae species (*Danafungia scruposa*, *Fungia fungites*, *Pleuractis paumotensis*) with the small *H*. *cochlea* having slightly shorter mean ingestion durations and rejection times (5.03 ± 0.47 mins and 18.45 ± 1.04 mins respectively) than the three larger fungiids. There was however no significant difference found in the consumption state or ingestion time of prey items between the coral species suggesting that prey were equally palatable to all four coral species. Differences in consumption (p = 0.21), ingestion (p = 0.09) and rejection time (p = 0.09) were not found between the three mushroom coral species. No correlation was found in any of the three measures between mouth size, nor between total coral diameter, when compared to size of prey items. Finally, no significant differences between in-situ and ex-situ trials for ingestion times (p = 0.30), rejection times (p = 0.17) or consumption (p = 0.47) were found.

**Table 4:** Tests of significance and correlation between numerous variables with relation to consumption score, time to completed ingestion and time to completed rejection. Significant results were considered as those with *p* < 0.05 or r values > 0.5 with regards to correlation.

	Consumption	Ingestion	Rejection
Prey species	**p < 0.001**	**p < 0.001**	**p < 0.001**
Prey size class	**p < 0.001**	**p < 0.001**	p = 0.14
Fungiids vs *Heteropsammia*	p = 0.15	p = 0.13	**p < 0.05**
Fungiid species	p = 0.10	p = 0.21	p = 0.09
Maximal size	r = -0.34	r = 0.22	r = -0.07
Mouth size	r = -0.15	r = 0.05	r = 0.11
In/Ex situ	p = 0.47	p = 0.30	p = 0.17

**Fig 7:**
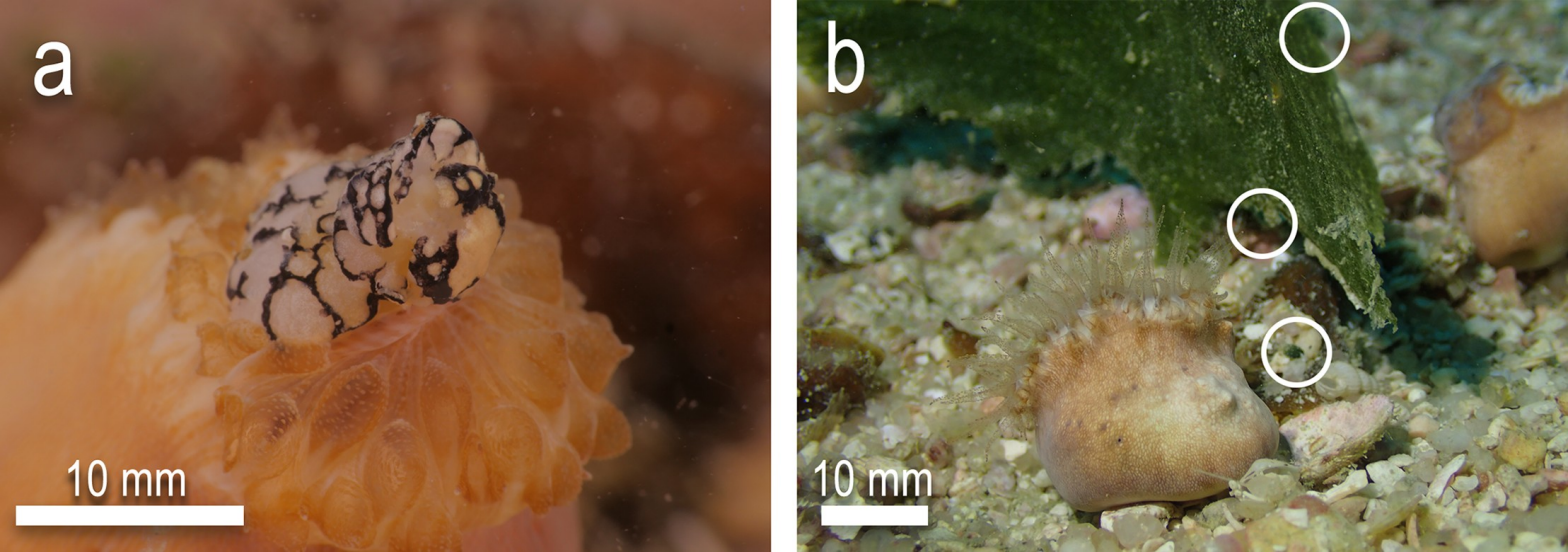
In-situ observations of natural predation of sea slugs by *Heteropsammia cochlea*. a) Partial ingestion of *Tubulophilinopsis pilsbryi* by *H*. *cochlea* b) *Heteropsammia cochlea* in close proximity to *Avrainvillea erecta*, hosting multiple *Costasiella* individuals (circled, partially visible).

During the study, additional observations were made of wild *H*. *cochlea* individuals ingesting wild sea slugs (Fig 7). An individual *Costasiella usagi* was observed to be caught in the tentacles of a coral and was completely ingested within 1.5 min. No rejection was observed after 30 mins. Additionally, a single individual of the cephalaspidean *Tubulophilinopsis pilsbryi* was observed partially ingested by *H*. *cochlea*, however it is not known if ingestion was completed and the prey consumed in this observation.

### Prey transport

Experimental transport rates of prey sea slugs upon mushroom corals showed no reliably discernible pattern with most prey successfully being transported by coral tentacles at rates between 10-40mm per minute (Table 5). The mean maximum diameter of mushroom corals used throughout the experiments was 105 mm. Mean rates of prey transport upon mushroom corals during in-situ observations were at 14.4 mm.min^-1^ and during ex-situ observations at 12 mm.min^-1^. During prey transport experiments, no significant transport rate difference (p = 0.41) was found between the prey species *P*. cf. *ocellatus*, *C*. *usagi*, and *C*. cf. *kuroshimae* in any of the coral species. Additionally, no difference (p = 0.37) was found in the transport rates of any sacoglossan species between the three fungiid corals. Finally, no significant difference (p = 0.70) was found in rate of transport on prey items placed on inner, middle or outer parts of the corals (see S5 Table).

**Table 5:** Mean rates of transport of prey items to the mouth of different mushroom coral species. All different prey species were tested upon all predator coral species. Rates shown include both in-situ and ex-situ trials.

**Prey species**	**Mean rate (**mm/min)
*Costasiella usagi*	16.75 ± 3.27 (n = 6)
*Plakobranchus* cf. *ocellatus*	8.47 ± 1.05 (n = 14)
*Elysia pusilla*	12.83 ± 1.91 (n = 6)
*Elysia* cf. *japonica*	11.72 ± 2.09 (n = 4)
*Costasiella* cf. *kuroshimae*	21.07 ± 7.31 (n = 7)
**Predator species**	**Mean rate per cm (**mm/min)
*Danafungia scruposa*	11.21 ± 1.99 (n = 12)
*Fungia fungites*	13.32 ± 1.79 (n = 10)
*Pleuractis paumotensis*	14.85 ± 3.69 (n = 15)
**Coral placement**	**Mean rate (**mm/min)
Inner	14.20 ± 4.55 (n = 12)
Middle	13.11 ± 1.84 (n = 11)
Outer	12.55 ± 1.88 (n = 14)

## Discussion

### Prey organisms

Based on the 240 trials, it was found that prey species and prey size were the most influential factors in determining the degree of consumption, duration of ingestion and length of time until rejection (Table 4). Across the six species of sea slugs tested, palatability of prey items (as inferred by consumption and rejection values) appeared to vary strongly. However, three species were preferentially predated by the corals, with individuals of both *Costasiella* species and *E*. *pusilla* all being rejected less than 25% of the time. Significantly higher mean consumption scores were also attributed to these same species. One distinctive attribute was found to be shared between these three species, when comparing biological, environmental and ecological factors of all six species. This being that all are strongly associated with their host algae, with 100% of individuals found found exclusively upon their algal prey. The remaining three species (*Plakobranchus* spp. and *E*. cf. *japonica*) are motile grazers, generalists feeding on filamentous algal prey from the substrate, which may have generated a selective pressure towards the development of more efficient chemical defenses against a wide range of predators present in the environment, not necessary in the case of the host-specific sacoglossans. This however requires further investigation.

The observation of a naturally caught and ingested *Costasiella usagi* was accompanied by multiple observations throughout the survey period of predatory *H*. *cochlea* individuals close by or in direct contact with numerous *Avrainvillea erecta* algae (Fig 7B) (the main algal prey for both *Costasiella* species at Koh Tao), several hosting their own predatory *Costasiella* spp. Mechanisms by which prey items are captured are not yet documented, however given the regular observation of *H*. *cochlea* individuals in contact with host algae of both *Costasiella* species, it is likely that some prey items may come in contact with the tentacles, and thus the cnidae of predatory corals, as was observed in the single instance of natural predation. These are the first recorded observations of sea slugs being ingested by *H*. *cochlea* under natural conditions (i.e. not part of feeding trial experiments). All individuals of *E*. *pusilla* were also found upon the green host algae *Halimeda macroloba*. At Chalok Bay, *H*. *macroloba*. can be found in dense but sporadic patches in shallow soft sediments with limited scleractinian presence, though fungiid and *Porites* corals have been observed nearby. However, *H*. *macroloba* is also found at deeper soft sediment habitats beyond the reef slope which are within the range of both fungiid and *H*. *cochlea* predators, though observations of either in the vicinity of *H*. *macroloba* are limited. The host specificity of slugs such as *C*. *usagi*, *C*. cf. *kuroshimae* and *E*. *pusilla* is likely to make them more vulnerable to capture due to a) being more accessible by predatory corals due to being elevated off the benthos and b) stationary relative to more mobile taxa such as *E*. cf. *japonica*, *P*. cf. *ocellatus* and *P*. cf. *papua*, therefore being less able to escape.

Several studies have been conducted investigating the species-specific defensive capacity in sacoglossans [54–56]. In all these cases however, the deterrent capabilities of sea slugs were tested largely on fish and a few non-cnidarian invertebrates. Given the present findings, it is possible that the compounds that are unpalatable or noxious to other animals, do not necessarily have the same effect on some scleractinian corals. Little is currently known about the diet or defensive capabilities of *Elysia* cf. *japonica* or *Plakobranchus papua*, however *P*. *ocellatus* (or more likely, the species that compose the *P*. *ocellatus* complex) is known to feed on at least five different species belonging to the green algae class Ulvophyceae [57]. A wide range of metabolites have been isolated from species of the *P*. *ocellatus* complex [56,58,59], however clarity on prey specificity and the resulting secondary metabolites in each case is needed to evaluate their defensive capability.

Upon extraction or rejection of all individuals in the non-consumed prey items, at the conclusion of the observation period, it was additionally recorded that all individuals of both *Plakobranchus* species and many individuals of *E*. cf. *japonica* were encased in a layer of mucus. It is likely that this enclosure of mucus was produced by the prey items upon ingestion as a possible means of protecting themselves from tissue degradation, presumably by creating a barrier of sorts against enzymatic activity while simultaneously reinforcing the production of unpalatable compounds. Although the production of unpalatable compounds in the mucus of sacoglossans is well documented [2,60], more specific studies with a particular focus on corals and other anthozoans should be carried out to test these theories.

Significant results were found regarding the role of prey size consumption and time of ingestion and rejection. Larger prey items, in particular those larger than 12 mm, were less regularly consumed and more rapidly rejected. However, it does appear that prey size is less of determining factor than prey species regarding preference of corals. For example, even the largest individual of the most preferred prey (*E*. *pusilla*) at 21 mm length was consumed readily, whereas the smallest individuals of *Plakobranchus* spp., were rejected (11 mm for *P*. *papua* and 8 mm for *P*. *ocellatus*). Likewise, the smallest individuals of *E*. cf. *japonica* were rejected, at 4 mm length, and all non-rejected individuals of *E*. cf. *japonica* covered the majority of the size range of the species, from 5–12 mm total length. Further investigations over broader prey species and wider size ranges are needed to more completely highlight the role and preference of prey size for scleractinian corals.

### Predators

Throughout the study, the data has shown minimal variation in ingestion times of prey items among the four different predators, implying a shared capacity for opportunistic ingestion in the corals. Likewise, differences in consumption score between the fungiids and *H*. *cochlea* were not significant suggesting that prey preference is shared among the scleractinian corals. This, however, does assume that preferences in large-mouthed, free-living corals are representative of a larger trend. While differences in rejection time were similar among the fungiids, it was found that *H*. *cochlea* was significantly quicker in rejecting unwanted prey items than the fungiids, which took approximately 20% longer. Thus, while all four corals may share preferences for certain prey pecies, this does not appear to translate to a shared tolerance and/or resilience to chemical deterrents in the prey. In evaluating the effectiveness and representation of ex-situ trials, data for all three metrics (consumption score, ingestion time and rejection time) were compared between all trials conducted on the reef and all conducted in aquaria. No significant differences were found between ingestion time, rejection time and consumption score (Tables 3–4, Fig 6C).

Not shown in the data are the 27 attempted in-situ trials where data had to be abandoned due to interactions from other marine life. During all these in-situ trials in assessing prey transport rates among corals and slugs, instances of prey capture, ingestion, and rejection by the moon wrasse *Thalassoma lunare* were observed. These instances were limited to smaller prey items (approx. 10–15 mm) and occurred by direct predation of prey while caught in the tentacle, or mouth, of the mushroom coral. This was not observed in trials with *H*. *cochlea* due to the relative absence of reef-associated fish from the soft-sediment habitats studied. These observations are examples of kleptoparasitism [61,62]. Kleptoparasitism on coral polyps is documented by acoelomorph and polychaete worms [63–66], and a variant of this feeding behaviour has been documented by nudibranchs feeding on hydroids [67]. In seven instances of kleptoparasitic behaviour by the wrasse, ingested prey were observed to be rejected shortly after capture, with the prey item falling on top of another mushroom coral and being readily ingested. This agrees with the observations of Hay and coworkers [55,68] regarding palatability and ingestion behaviour of *Costasiella ocellifera* and *T*. *lunare*. It can therefore be suggested that one of the mechanisms by which predatory mushroom corals capture most benthic prey is by the temporary elevation of rejected prey and the opportunistic capture of prey. This further works to highlight the complexity that drives the benthic trophic system, such as those of corals in coral reefs, which had till now only been known to capture and ingest zooplankton.

Outside of the extremely uncommon scenario that prey items are released or captured directly in the mouth, the majority of prey capture in fungiid corals happens at the numerous tentacles among the long septa. The capture and transport of food by fungiid corals was investigated by Abe [69] however rates of transport were not investigated. Therefore, the present study sheds light on the transport rates of captured sea slug prey across the coral disc to the mouth in the three fungiids. Rates of transport remained consistent across three species of varying sizes and across the three species of coral. This was also the case when investigating if placement upon the coral (closer or further from the mouth) had an impact. All in-situ prey-transport trials were conducted during calm conditions with low wind speed or wave action (no quantitative data was however collected on these specific variables). The transport of prey items was done so by wave motion transferring prey from one tentacle to the next, before arriving at the mouth, and appeared to depend on the regularity and minimum force upon the coral surface. This is supported by the finding of ex-situ replications of prey transport that were carried out by manually mimicking wave action and resulting in mean transport of 12 mm.min^-1^, whereas in-situ transport occurred at 14.4 mm.min^-1^. Occasions in which strong wave action inhibited successful transport of captured prey often resulted in the loss of prey items. This agrees with the observation of prey transport in salps by mushroom corals [25] suggesting that strong wave motion negatively impacts prey retention and transport. However, it does appear that a minimal threshold of motion is also needed to promote transport, which also minimises opportunistic prey capture by kleptopredators.

### Habitat and behaviour

**Fig 8:**
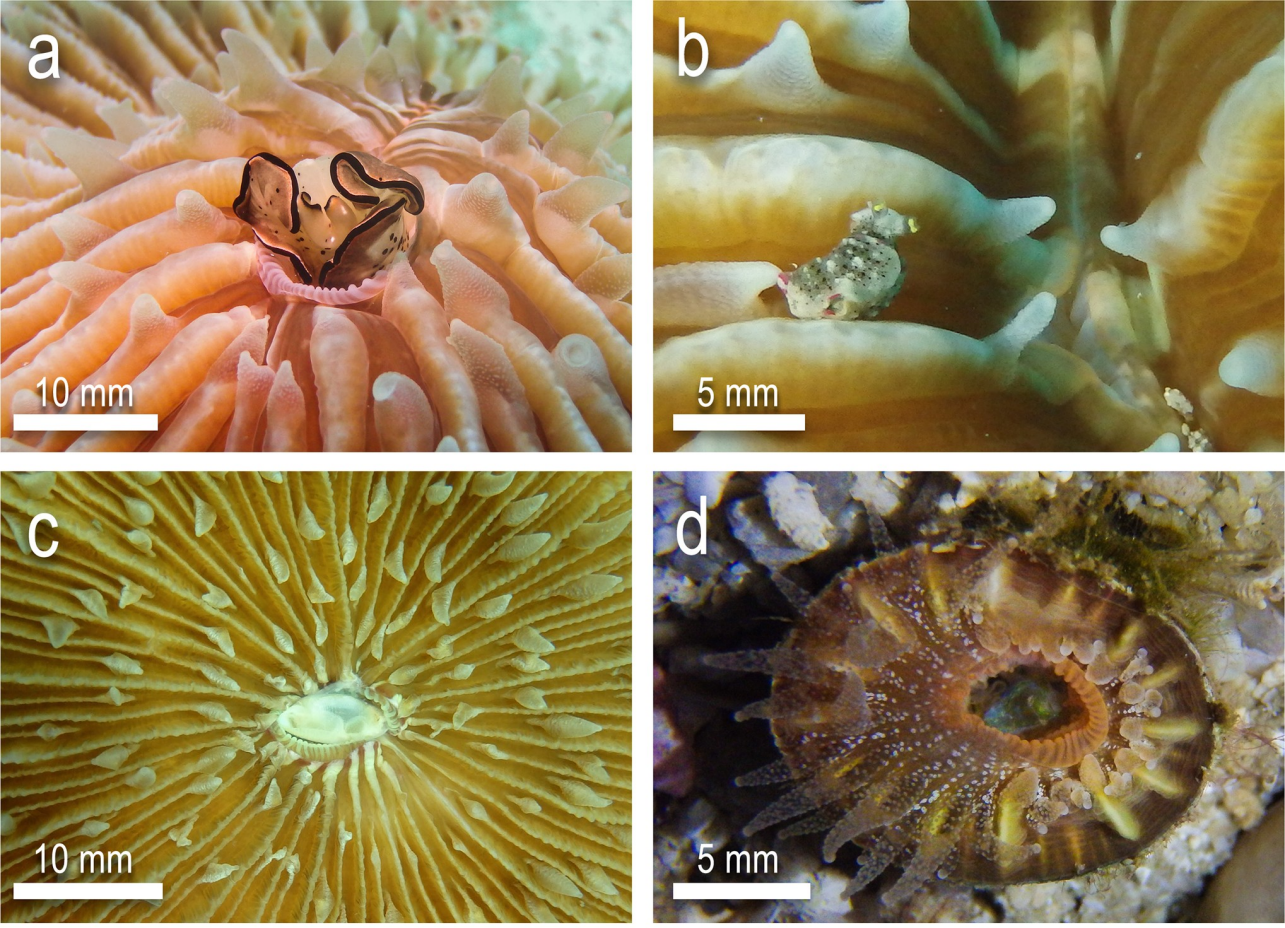
Examples of additional test trials with a) *Elysia marginata* and *Fungia fungites*, b) *Elysia asbecki* with *F*. *fungites*, c) *Aliculastrum debile* with *F*. *fungites* and d) *Elysia pusilla* with *Truncatoflabellum* sp.

Not included in the analyses were the trials of a number of other sea slug and coral taxa (Fig 8) including the sacoglossans *Elysia asbecki* and *Elysia marginata*, the cephalaspidean *Aliculastrum debile* and the nudibranch *Dermatobranchus striatus*. A number of other free-living coral taxa were also investigated as part of a pilot investigation, including *Truncatoflabellum* sp. (ex situ only) and *Heterocyathus aequicostatus* (both in-situ and ex-situ) that were collected at the same locations and depth profiles as *H*. *cochlea*. Due to the limited number of trials conducted, these results were excluded from the statistical tests. Nonetheless, observational data indicated that both *E*. *asbecki* and *E*. *marginata*, were not palatable to mushroom coral taxa, and were rejected within 15–20 min post ingestion, after which all animals crawled away with minimal indication of tissue loss. Individuals of *A*. *debile* were only partially ingested and rapidly rejected during trials with *F*. *fungites* and *P*. *paumotensis*. This may be due to the presence of the relatively large external shell, which is internalised or completely absent in many other sea slug taxa such as those in the present study. This shell may have proven too large or rigid to be manipulated inside the mouth of the coral. It was noted however that individuals of *D*. *striatus* were completely ingested, and often consumed by the corals *F*. *fungites* and *P*. *paumotensis*, and the soft-sediment corals *H*. *cochlea*, *H*. *aequiocostatus* and *Truncatoflabellum* sp. Complete ingestion and partial or complete consumption was also recorded in the majority of individuals of *C*. cf. *kuroshimae*, *C*. *usagi* and *E*. *pusilla* when applied to *Truncatoflabellum* sp. and *H*. *aequiocostatus*. Individuals of *Elysia* cf. *japonica* were rejected intact when fed to *H*. *aequiocostatus* but were partially or complete consumed when fed to *Truncatoflabellum* sp. Though these observations are intriguing and provide further dimensions to the present investigation, more replications and focus on these taxa are required to add support to understanding these behavioural traits.

*Heteropsammia* spp. are distributed along the shallow waters of the Indo-Pacific [29,31,70], but at Koh Tao, are found exclusively in the deeper soft-sediment habitats, alongside *Truncatoflabellum* sp. and *H*. *aequiocostatus*. Additionally, three of the prey species used in the trials are known to be exclusive to the deeper soft sediment habitats of Koh Tao [44]. While no significant differences were found when comparing consumption, ingestion or rejection between soft sediment associated prey or predators to their reef associated counterparts, a combination of natural and experimental observations documented here now identify two ecologically distinct types of scleractinian coral capable of predation upon sea slugs. Beyond the fact that widespread predators of sea slugs may have potentially been hiding in plain sight, it is worth noting that the long-standing convention that scleractinian corals predate exclusively on planktonic or demersal prey, or particulate and dissolved organic matter, is no longer viable.

## Supporting information

S1 Table Complete raw data of trials conducted, as separated by prey species. PIOL values indicate Post Ingestion Observation Length in minutes. Consumption scores range from 0 indicating no visible tissue degradation to 1 indicating heavy tissue degradation.
(DOCX)

S2 Table Mean ingestion duration ± standard error (min) for each predator-prey trial. (DOCX)

S3 Table Mean time till rejection ± standard error (min) for each predator-prey trial. (DOCX)

S4 Table Mean consumption score ± standard error for each predator-prey trial. (DOCX)

S5 Table Mean rate of prey transport ± standard error (mm.min^-1^) for all prey and predators tested. (DOCX)
