# Supplementary material for: Selective consumption of sacoglossan sea slugs (Mollusca: Gastropoda) by scleractinian corals (Cnidaria: Anthozoa)
Source: PLoS One. 2019 Apr 29;14(4):e0215063. doi: 10.1371/journal.pone.0215063 (PMC6488191; doi:10.1371/journal.pone.0215063)
Supplement: S1 Table — PIOL values indicate Post Ingestion Observation Length in minutes. Consumption scores range from 0 indicating no visible tissue degradation to 1 indicating heavy tissue degradation. (DOCX) [file pone.0215063.s001.docx]

| ***Plakobranchus* cf. *papua*** | | | | | | | | | | | |
| --- | --- | --- | --- | --- | --- | --- | --- | --- | --- | --- | --- |
| **Trial Type** | **Prey Species** | **Predator Species** | **Prey Length (mm)** | **Mouth Length (mm)** | **Coral Diameter (mm)** | **Time on coral (mins)** | **Time at mouth (mins)** | **Ingestion Completed (mins)** | **Rejection Complete (mins)** | **P.I.O.L (mins)** | **Consumption Score** |
| in situ | *Plakobranchus* cf. *papua* | *Pleuractis paumotensis* | 12 | 23 | 205 | 0 | 0 | 12 | 24 | - | 0 |
| in situ | *Plakobranchus* cf. *papua* | *Fungia fungites* | 14 | 28 | 195 | 0 | 0 | 4 | 20 | - | 0 |
| in situ | *Plakobranchus* cf. *papua* | *Danafungia scruposa* | 31 | 29 | 300 | 0 | 0 | Incomplete Ingestion | 8 | - | 0 |
| in situ | *Plakobranchus* cf. *papua* | *Pleuractis paumotensis* | 28 | 30 | 225 | 0 | 0 | 9 | 15 | - | 0 |
| in situ | *Plakobranchus* cf. *papua* | *Danafungia scruposa* | 19 | 31 | 255 | 0 | 0 | 9 | 17 | - | 0 |
| in situ | *Plakobranchus* cf. *papua* | *Pleuractis paumotensis* | 24 | 33 | 200 | 0 | 0 | 10 | 22 | - | 0 |
| in situ | *Plakobranchus* cf. *papua* | *Danafungia scruposa* | 26 | 29 | 290 | 0 | 0 | 6 | 12 | - | 0 |
| in situ | *Plakobranchus* cf. *papua* | *Fungia fungites* | 29 | 20 | 240 | 0 | 0 | 5 | 13 | - | 0 |
| in situ | *Plakobranchus* cf. *papua* | *Pleuractis paumotensis* | 26 | 38 | 190 | 0 | 0 | 8 | 27 | - | 0 |
| in situ | *Plakobranchus* cf. *papua* | *Pleuractis paumotensis* | 33 | 27 | 150 | 0 | 0 | 2 | - | 65 | 0 |
| ex situ | *Plakobranchus* cf. *papua* | *Pleuractis paumotensis* | 26 | 35 | 245 | 0 | 0 | 9 | 17 | - | 0 |
| ex situ | *Plakobranchus* cf. *papua* | *Pleuractis paumotensis* | 20 | 30 | 240 | 0 | 0 | Incomplete Ingestion | 18 | - | 0 |
| ex situ | *Plakobranchus* cf. *papua* | *Pleuractis paumotensis* | 28 | 29 | 160 | 0 | 0 | 5 | - | 74 | 0 |
| ex situ | *Plakobranchus* cf. *papua* | *Pleuractis paumotensis* | 19 | 24 | 235 | 0 | 0 | 3 | 21 | - | 0 |
| ex situ | *Plakobranchus* cf. *papua* | *Pleuractis paumotensis* | 19 | 29 | 225 | 0 | 0 | 12 | - | 88 | 0 |
| ex situ | *Plakobranchus* cf. *papua* | *Pleuractis paumotensis* | 15 | 33 | 275 | 0 | 0 | 6 | 12 | - | 0 |
| ex situ | *Plakobranchus* cf. *papua* | *Pleuractis paumotensis* | 25 | 25 | 270 | 0 | 0 | 4 | 10 | - | 0 |
| ex situ | *Plakobranchus* cf. *papua* | *Pleuractis paumotensis* | 30 | 30 | 140 | 0 | 0 | 4 | 19 | - | 0 |
| ex situ | *Plakobranchus* cf. *papua* | *Pleuractis paumotensis* | 27 | 31 | 195 | 0 | 0 | 2 | 14 | - | 0 |
| ex situ | *Plakobranchus* cf. *papua* | *Pleuractis paumotensis* | 30 | 31 | 180 | 0 | 0 | 1 | 6 | - | 0 |
| in situ | *Plakobranchus* cf. *papua* | *Heteropsammia cochlea* | 29 | 26 | 27 | 0 | 0 | Incomplete Ingestion | 7 | - | 0 |
| in situ | *Plakobranchus* cf. *papua* | *Heteropsammia cochlea* | 18 | 29 | 30 | 0 | 0 | Incomplete Ingestion | 22 | - | 0 |
| in situ | *Plakobranchus* cf. *papua* | *Heteropsammia cochlea* | 22 | 30 | 31 | 0 | 0 | Incomplete Ingestion | 8 | - | 0 |
| in situ | *Plakobranchus* cf. *papua* | *Heteropsammia cochlea* | 20 | 28 | 30 | 0 | 0 | Incomplete Ingestion | 4 | - | 0 |
| in situ | *Plakobranchus* cf. *papua* | *Heteropsammia cochlea* | 20 | 31 | 31 | 0 | 0 | Incomplete Ingestion | 12 | - | 0 |
| in situ | *Plakobranchus* cf. *papua* | *Heteropsammia cochlea* | 25 | 29 | 30 | 0 | 0 | Incomplete Ingestion | - | - | 0 |
| in situ | *Plakobranchus* cf. *papua* | *Heteropsammia cochlea* | 27 | 28 | 25 | 0 | 0 | Incomplete Ingestion | 8 | - | 0 |
| in situ | *Plakobranchus* cf. *papua* | *Heteropsammia cochlea* | 28 | 22 | 25 | 0 | 0 | 16 | - | 65 | 0 |
| in situ | *Plakobranchus* cf. *papua* | *Heteropsammia cochlea* | 29 | 22 | 26 | 0 | 0 | Incomplete Ingestion | - | - | 0 |
| in situ | *Plakobranchus* cf. *papua* | *Heteropsammia cochlea* | 18 | 23 | 22 | 0 | 0 | Incomplete Ingestion | 6 | - | 0 |
| ex situ | *Plakobranchus* cf. *papua* | *Heteropsammia cochlea* | 32 | 26 | 26 | 0 | 0 | 7 | 21 | - | 0 |
| ex situ | *Plakobranchus* cf. *papua* | *Heteropsammia cochlea* | 25 | 25 | 27 | 0 | 0 | Incomplete Ingestion | - | - | 0 |
| ex situ | *Plakobranchus* cf. *papua* | *Heteropsammia cochlea* | 28 | 26 | 29 | 0 | 0 | Incomplete Ingestion | - | - | 0 |
| ex situ | *Plakobranchus* cf. *papua* | *Heteropsammia cochlea* | 20 | 22 | 24 | 0 | 0 | Incomplete Ingestion | - | - | 0 |
| ex situ | *Plakobranchus* cf. *papua* | *Heteropsammia cochlea* | 11 | 29 | 32 | 0 | 0 | Incomplete Ingestion | - | - | 0 |
| ex situ | *Plakobranchus* cf. *papua* | *Heteropsammia cochlea* | 17 | 28 | 27 | 0 | 0 | Incomplete Ingestion | - | - | 0 |
| ex situ | *Plakobranchus* cf. *papua* | *Heteropsammia cochlea* | 25 | 27 | 29 | 0 | 0 | Incomplete Ingestion | - | - | 0 |
| ex situ | *Plakobranchus* cf. *papua* | *Heteropsammia cochlea* | 25 | 26 | 29 | 0 | 0 | Incomplete Ingestion | - | - | 0 |
| ex situ | *Plakobranchus* cf. *papua* | *Heteropsammia cochlea* | 24 | 28 | 28 | 0 | 0 | Incomplete Ingestion | - | - | 0 |
| ex situ | *Plakobranchus* cf. *papua* | *Heteropsammia cochlea* | 18 | 27 | 28 | 0 | 0 | Incomplete Ingestion | 8 | - | 0 |
| ***Elysia pusilla*** | | | | | | | | | | | |
| **Trial Type** | **Prey Species** | **Predator Species** | **Prey Length (mm)** | **Mouth Length (mm)** | **Coral Diameter (mm)** | **Time on coral (mins)** | **Time at mouth (mins)** | **Ingestion Completed (mins)** | **Rejection Complete (mins)** | **P.I.O.L (mins)** | **Consumption Score** |
| in situ | *Elysia pusilla* | *Pleuractis paumotensis* | 12 | 14 | 180 | 0 | 2 | 3 | - | 43 | - |
| in situ | *Elysia pusilla* | *Pleuractis paumotensis* | 10 | 21 | 175 | 0 | 6 | 8 | - | 41 | - |
| in situ | *Elysia pusilla* | *Pleuractis paumotensis* | 14 | 33 | 165 | 0 | 2 | 6 | - | 55 | - |
| in situ | *Elysia pusilla* | *Fungia fungites* | 21 | 25 | 205 | 0 | 3 | 10 | - | 45 | - |
| in situ | *Elysia pusilla* | *Danafungia scruposa* | 9 | 23 | 80 | 0 | 4 | 5 | - | 70 |  |
| in situ | *Elysia pusilla* | *Pleuractis paumotensis* | 10 | 40 | 250 | 0 | 0 | 4 | - | 69 | 0.5 |
| in situ | *Elysia pusilla* | *Pleuractis paumotensis* | 8 | 40 | 155 | 0 | 0 | 4 | - | 93 | 0.5 |
| in situ | *Elysia pusilla* | *Fungia fungites* | 12 | 44 | 250 | 0 | 0 | 19 | - | 59 | 1 |
| in situ | *Elysia pusilla* | *Fungia fungites* | 8 | 32 | 175 | 0 | 0 | 1 | - | 74 | 1 |
| in situ | *Elysia pusilla* | *Fungia fungites* | 9 | 20 | 122 | 0 | 0 | 7 | - | 88 | 1 |
| ex situ | *Elysia pusilla* | *Fungia fungites* | 7 | 17 | 140 | 0 | 2 | 3 | - | 120 | - |
| ex situ | *Elysia pusilla* | *Pleuractis paumotensis* | 12 | 26 | 110 | 0 | 0 | 2 | - | 180 | 0.5 |
| ex situ | *Elysia pusilla* | *Danafungia scruposa* | 7 | 14 | 115 | 0 | 0 | 1 | - | 180 | 1 |
| ex situ | *Elysia pusilla* | *Danafungia scruposa* | 16 | 15 | 90 | 0 | 0 | 2 | - | 240 | 1 |
| ex situ | *Elysia pusilla* | *Danafungia scruposa* | 8 | 12 | 95 | 0 | 0 | 1 | - | 180 | - |
| ex situ | *Elysia pusilla* | *Fungia fungites* | 10 | 24 | 155 | 0 | 0 | 5 | - | 125 | 0.5 |
| ex situ | *Elysia pusilla* | *Fungia fungites* | 6 | 18 | 115 | 0 | 0 | 12 | - | 120 | 0.5 |
| ex situ | *Elysia pusilla* | *Fungia fungites* | 6 | 21 | 122 | 0 | 0 | 5 | - | 120 | 0.5 |
| ex situ | *Elysia pusilla* | *Fungia fungites* | 8 | 8 | 35 | 0 | 0 | 20 | - | 120 | 0.5 |
| ex situ | *Elysia pusilla* | *Fungia fungites* | 7 | 16 | 35 | 0 | 0 | 2 | - | 90 | 0.5 |
| in situ | *Elysia pusilla* | *Heteropsammia cochlea* | 8 | 20 | 26 | 0 | 0 | 1 | - | 56 | 0.5 |
| in situ | *Elysia pusilla* | *Heteropsammia cochlea* | 8 | 22 | 22 | 0 | 0 | 6 | - | 71 | 1 |
| in situ | *Elysia pusilla* | *Heteropsammia cochlea* | 7 | 21 | 27 | 0 | 0 | 4 | - | 47 | 1 |
| in situ | *Elysia pusilla* | *Heteropsammia cochlea* | 5 | 16 | 18 | 0 | 0 | 2 | - | 48 | 0.5 |
| in situ | *Elysia pusilla* | *Heteropsammia cochlea* | 6 | 24 | 26 | 0 | 0 | 5 | - | 34 | 0.5 |
| in situ | *Elysia pusilla* | *Heteropsammia cochlea* | 8 | 22 | 28 | 0 | 0 | 7 | - | 30 | 0.5 |
| in situ | *Elysia pusilla* | *Heteropsammia cochlea* | 9 | 16 | 22 | 0 | 0 | 7 | - | 27 | 0.5 |
| in situ | *Elysia pusilla* | *Heteropsammia cochlea* | 7 | 28 | 20 | 0 | 0 | 8 | - | 42 | 1 |
| in situ | *Elysia pusilla* | *Heteropsammia cochlea* | 6 | 20 | 26 | 0 | 0 | 21 | - | 28 | 0.5 |
| in situ | *Elysia pusilla* | *Heteropsammia cochlea* | 5 | 22 | 22 | 0 | 0 | 24 | - | 38 | 0 |
| ex situ | *Elysia pusilla* | *Heteropsammia cochlea* | 6 | 24 | 27 | 0 | 0 | 9 | - | 61 | 0 |
| ex situ | *Elysia pusilla* | *Heteropsammia cochlea* | 7 | 27 | 28 | 0 | 0 | 4 | - | 165 | 1 |
| ex situ | *Elysia pusilla* | *Heteropsammia cochlea* | 2 | 26 | 32 | 0 | 0 | 5 | - | 120 | 1 |
| ex situ | *Elysia pusilla* | *Heteropsammia cochlea* | 8 | 23 | 23 | 0 | 0 | 18 | - | 77 | - |
| ex situ | *Elysia pusilla* | *Heteropsammia cochlea* | 10 | 27 | 28 | 0 | 0 | 27 | - | 150 | - |
| ex situ | *Elysia pusilla* | *Heteropsammia cochlea* | 5 | 24 | 26 | 0 | 0 | 3 | - | 120 | 1 |
| ex situ | *Elysia pusilla* | *Heteropsammia cochlea* | 8 | 26 | 22 | 0 | 0 | 1 | - | 155 | 0.5 |
| ex situ | *Elysia pusilla* | *Heteropsammia cochlea* | 6 | 22 | 24 | 0 | 0 | 6 | - | 360 | 1 |
| ex situ | *Elysia pusilla* | *Heteropsammia cochlea* | 3 | 22 | 26 | 0 | 0 | 4 | - | 120 | 0.5 |
| ex situ | *Elysia pusilla* | *Heteropsammia cochlea* | 4 | 30 | 30 | 0 | 0 | 2 | 18 | - | 0.5 |
| ***Costasiella usagi*** | | | | | | | | | | | |
| **Trial Type** | **Prey Species** | **Predator Species** | **Prey Length (mm)** | **Mouth Length (mm)** | **Coral Diameter (mm)** | **Time on coral (mins)** | **Time at mouth (mins)** | **Ingestion Completed (mins)** | **Rejection Complete (mins)** | **P.I.O.L (mins)** | **Consumption Score** |
| in situ | *Costasiella usagi* | *Fungia fungites* | 8 | 21 | 295 | 0 | 0 | 1 | - | 60 | 0.5 |
| in situ | *Costasiella usagi* | *Fungia fungites* | 6 | 28 | 150 | 0 | 0 | 2 | - | 52 | 0 |
| in situ | *Costasiella usagi* | *Pleuractis paumotensis* | 6 | 24 | 180 | 0 | 4 | 6 | - | 50 |  |
| in situ | *Costasiella usagi* | *Pleuractis paumotensis* | 5 | 24 | 210 | 0 | 0 | 1 | - | 60 | 0.5 |
| in situ | *Costasiella usagi* | *Pleuractis paumotensis* | 4 | 21 | 280 | 0 | 5 | 6 | - | 60 |  |
| in situ | *Costasiella usagi* | *Danafungia scruposa* | 2 | 45 | 120 | 0 | 0 | 2 | - | 81 | 0.5 |
| in situ | *Costasiella usagi* | *Fungia fungites* | 4 | 35 | 290 | 0 | 0 | 3 | - | 60 | 0.5 |
| in situ | *Costasiella usagi* | *Fungia fungites* | 4 | 35 | 160 | 0 | 0 | 1 | 18 | - | 0 |
| in situ | *Costasiella usagi* | *Fungia fungites* | 3 | 20 | 240 | 0 | 4 | 5 | - | 90 | 0 |
| in situ | *Costasiella usagi* | *Danafungia scruposa* | 2 | 34 | 180 | 0 | 6 | 7 | - | 90 | 1 |
| ex situ | *Costasiella usagi* | *Danafungia scruposa* | 4 | 31 | 180 | 0 | 0 | 4 | - | 120 | - |
| ex situ | *Costasiella usagi* | *Danafungia scruposa* | 6 | 29 | 165 | 0 | 1 | 3 | - | 180 | - |
| ex situ | *Costasiella usagi* | *Fungia fungites* | 5 | 42 | 310 | 0 | 6 | 7 | - | 180 | - |
| ex situ | *Costasiella usagi* | *Danafungia scruposa* | 3 | 18 | 225 | 0 | 0 | 4 | - | 180 | - |
| ex situ | *Costasiella usagi* | *Danafungia scruposa* | 6 | 22 | 275 | 0 | 0 | 1 | - | 180 | - |
| ex situ | *Costasiella usagi* | *Fungia fungites* | 3 | 26 | 118 | 0 | 0 | 2 | 6 | - | 0 |
| ex situ | *Costasiella usagi* | *Danafungia scruposa* | 1 | 15 | 75 | 0 | 0 | 5 | - | 90 | 0.5 |
| ex situ | *Costasiella usagi* | *Fungia fungites* | 3 | 22 | 125 | 0 | 0 | 4 | 24 | - | 0 |
| ex situ | *Costasiella usagi* | *Danafungia scruposa* | 3 | 25 | 140 | 0 | 0 | 6 | - | 90 | 1 |
| ex situ | *Costasiella usagi* | *Danafungia scruposa* | 4 | 8 | 35 | 0 | 0 | 1 | - | 120 | 1 |
| in situ | *Costasiella usagi* | *Heteropsammia cochlea* | 4 | 22 | 22 | 0 | 0 | 2 | - | 35 | 0.5 |
| in situ | *Costasiella usagi* | *Heteropsammia cochlea* | 3 | 28 | 28 | 0 | 0 | 3 | - | 33 | 0 |
| in situ | *Costasiella usagi* | *Heteropsammia cochlea* | 3 | 24 | 26 | 0 | 0 | 5 | - | 25 | 0.5 |
| in situ | *Costasiella usagi* | *Heteropsammia cochlea* | 4 | 24 | 20 | 0 | 0 | 3 | 9 | - | 0 |
| in situ | *Costasiella usagi* | *Heteropsammia cochlea* | 3 | 25 | 30 | 0 | 0 | 2 | - | 90 | 0.5 |
| in situ | *Costasiella usagi* | *Heteropsammia cochlea* | 4 | 21 | 22 | 0 | 0 | 2 | - | 90 | 0.5 |
| in situ | *Costasiella usagi* | *Heteropsammia cochlea* | 2 | 12 | 22 | 0 | 0 | 1 | - | 60 | 0 |
| in situ | *Costasiella usagi* | *Heteropsammia cochlea* | 3 | 31 | 27 | 0 | 0 | 2 | - | 87 | 0.5 |
| in situ | *Costasiella usagi* | *Heteropsammia cochlea* | 2 | 16 | 20 | 0 | 0 | 1 | - | 46 | 0.5 |
| in situ | *Costasiella usagi* | *Heteropsammia cochlea* | 3 | 20 | 18 | 0 | 0 | 2 | - | 57 | 0.5 |
| ex situ | *Costasiella usagi* | *Heteropsammia cochlea* | 2 | 19 | 22 | 0 | 0 | 3 | - | 105 | 1 |
| ex situ | *Costasiella usagi* | *Heteropsammia cochlea* | 5 | 20 | 26 | 0 | 0 | 2 | 12 | - | 0 |
| ex situ | *Costasiella usagi* | *Heteropsammia cochlea* | 4 | 19 | 22 | 0 | 0 | 1 | 35 | - | 0 |
| ex situ | *Costasiella usagi* | *Heteropsammia cochlea* | 4 | 18 | 26 | 0 | 0 | 1 | - | 120 | 0.5 |
| ex situ | *Costasiella usagi* | *Heteropsammia cochlea* | 2 | 18 | 34 | 0 | 0 | 3 | - | 105 | 1 |
| ex situ | *Costasiella usagi* | *Heteropsammia cochlea* | 5 | 26 | 33 | 0 | 0 | 8 | 49 | - | 0.5 |
| ex situ | *Costasiella usagi* | *Heteropsammia cochlea* | 3 | 26 | 28 | 0 | 0 | 2 | 68 | - | 0.5 |
| ex situ | *Costasiella usagi* | *Heteropsammia cochlea* | 6 | 26 | 28 | 0 | 0 | 4 | - | 100 | 0 |
| ex situ | *Costasiella usagi* | *Heteropsammia cochlea* | 3 | 24 | 25 | 0 | 0 | 2 | - | 90 | 1 |
| ex situ | *Costasiella usagi* | *Heteropsammia cochlea* | 4 | 20 | 25 | 0 | 0 | 1 | - | 105 | 0.5 |
| ***Costasiella* cf. *kuroshimae*** | | | | | | | | | | | |
| **Trial Type** | **Prey Species** | **Predator Species** | **Prey Length (mm)** | **Mouth Length (mm)** | **Coral Diameter (mm)** | **Time on coral (mins)** | **Time at mouth (mins)** | **Ingestion Completed (mins)** | **Rejection Complete (mins)** | **P.I.O.L (mins)** | **Consumption Score** |
| in situ | *Costasiella* cf. *kuroshimae* | *Pleuractis paumotensis* | 6 | 17 | 130 | 0 | 3 | 4 | - | 45 | - |
| in situ | *Costasiella* cf. *kuroshimae* | *Pleuractis paumotensis* | 4 | 32 | 400 | 0 | 1 | 2 | 4 | - | 0 |
| in situ | *Costasiella* cf. *kuroshimae* | *Danafungia scruposa* | 4 | 15 | 215 | 0 | 2 | 3 | - | 55 | - |
| in situ | *Costasiella* cf. *kuroshimae* | *Pleuractis paumotensis* | 5 | 19 | 100 | 0 | 3 | 5 | - | 50 | - |
| in situ | *Costasiella* cf. *kuroshimae* | *Fungia fungites* | 5 | 35 | 365 | 0 | 8 | 9 | - | 60 | - |
| in situ | *Costasiella* cf. *kuroshimae* | *Pleuractis paumotensis* | 5 | 38 | 100 | 0 | 0 | 2 | - | 88 | 1 |
| in situ | *Costasiella* cf. *kuroshimae* | *Pleuractis paumotensis* | 5 | 34 | 86 | 0 | 0 | 3 | - | 78 | - |
| in situ | *Costasiella* cf. *kuroshimae* | *Danafungia scruposa* | 5 | 21 | 200 | 0 | 0 | 2 | - | 60 | 0.5 |
| in situ | *Costasiella* cf. *kuroshimae* | *Danafungia scruposa* | 5 | 13 | 130 | 0 | 0 | 3 | - | 57 | 0.5 |
| in situ | *Costasiella* cf. *kuroshimae* | *Danafungia scruposa* | 3 | 38 | 190 | 0 | 0 | 1 | - | 88 | 0.5 |
| ex situ | *Costasiella* cf. *kuroshimae* | *Danafungia scruposa* | 7 | 24 | 365 | 0 | 5 | 6 | - | 120 | - |
| ex situ | *Costasiella* cf. *kuroshimae* | *Danafungia scruposa* | 8 | 10 | 75 | 0 | 3 | 4 | - | 180 | 1 |
| ex situ | *Costasiella* cf. *kuroshimae* | *Fungia fungites* | 4 | 27 | 185 | 0 | 0 | 1 | - | 150 | 1 |
| ex situ | *Costasiella* cf. *kuroshimae* | *Danafungia scruposa* | 4 | 33 | 265 | 0 | 0 | 1 | - | 120 | - |
| ex situ | *Costasiella* cf. *kuroshimae* | *Danafungia scruposa* | 4 | 39 | 335 | 0 | 0 | 1 | - | 180 | - |
| ex situ | *Costasiella* cf. *kuroshimae* | *Fungia fungites* | 6 | 24 | 120 | 0 | 0 | 5 | - | 120 | 0.5 |
| ex situ | *Costasiella* cf. *kuroshimae* | *Fungia fungites* | 4 | 24 | 134 | 0 | 0 | 2 | - | 90 | 0 |
| ex situ | *Costasiella* cf. *kuroshimae* | *Fungia fungites* | 5 | 23 | 125 | 0 | 0 | 23 | - | 120 | 0 |
| ex situ | *Costasiella* cf. *kuroshimae* | *Fungia fungites* | 4 | 16 | 85 | 0 | 0 | 1 | - | 120 | 0.5 |
| ex situ | *Costasiella* cf. *kuroshimae* | *Fungia fungites* | 4 | 20 | 104 | 0 | 0 | 3 | 17 | - | 0 |
| in situ | *Costasiella* cf. *kuroshimae* | *Heteropsammia cochlea* | 5 | 18 | 19 | 0 | 0 | 4 | - | 67 | 0.5 |
| in situ | *Costasiella* cf. *kuroshimae* | *Heteropsammia cochlea* | 3 | 10 | 16 | 0 | 0 | 1 | - | 70 | 0.5 |
| in situ | *Costasiella* cf. *kuroshimae* | *Heteropsammia cochlea* | 7 | 24 | 34 | 0 | 0 | 19 | - | 79 | 0 |
| in situ | *Costasiella* cf. *kuroshimae* | *Heteropsammia cochlea* | 2.5 | 14 | 22 | 0 | 0 | 4 | - | 71 | 0.5 |
| in situ | *Costasiella* cf. *kuroshimae* | *Heteropsammia cochlea* | 2 | 23 | 32 | 0 | 0 | 1 | - | 90 | 1 |
| in situ | *Costasiella* cf. *kuroshimae* | *Heteropsammia cochlea* | 4 | 19 | 23 | 0 | 0 | 2 | - | 90 | 0.5 |
| in situ | *Costasiella* cf. *kuroshimae* | *Heteropsammia cochlea* | 6 | 26 | 24 | 0 | 0 | 1 | - | 51 | 0.5 |
| in situ | *Costasiella* cf. *kuroshimae* | *Heteropsammia cochlea* | 6 | 16 | 22 | 0 | 0 | 3 | - | 28 | 0.5 |
| in situ | *Costasiella* cf. *kuroshimae* | *Heteropsammia cochlea* | 6 | 20 | 22 | 0 | 0 | 1 | - | 59 | 0 |
| in situ | *Costasiella* cf. *kuroshimae* | *Heteropsammia cochlea* | 4 | 24 | 26 | 0 | 0 | 10 | - | 52 | 0 |
| ex situ | *Costasiella* cf. *kuroshimae* | *Heteropsammia cochlea* | 6 | 18 | 18 | 0 | 0 | 5 | - | 70 | 0 |
| ex situ | *Costasiella* cf. *kuroshimae* | *Heteropsammia cochlea* | 5 | 26 | 24 | 0 | 0 | 3 | 15 |  | 0 |
| ex situ | *Costasiella* cf. *kuroshimae* | *Heteropsammia cochlea* | 5 | 26 | 18 | 0 | 0 | 1 | - | 85 | 0.5 |
| ex situ | *Costasiella* cf. *kuroshimae* | *Heteropsammia cochlea* | 1 | 18 | 23 | 0 | 0 | 1 | 18 |  | 0 |
| ex situ | *Costasiella* cf. *kuroshimae* | *Heteropsammia cochlea* | 5 | 26 | 20 | 0 | 0 | 7 | - | 95 | 0 |
| ex situ | *Costasiella* cf. *kuroshimae* | *Heteropsammia cochlea* | 5.5 | 28 | 34 | 0 | 0 | 4 | - | 120 | 0 |
| ex situ | *Costasiella* cf. *kuroshimae* | *Heteropsammia cochlea* | 5 | 26 | 28 | 0 | 0 | 9 | - | 180 | 0.5 |
| ex situ | *Costasiella* cf. *kuroshimae* | *Heteropsammia cochlea* | 4 | 23 | 26 | 0 | 0 | 3 | - | 120 | 0.5 |
| ex situ | *Costasiella* cf. *kuroshimae* | *Heteropsammia cochlea* | 4 | 23 | 26 | 0 | 0 | 5 | 11 |  | 0.5 |
| ex situ | *Costasiella* cf. *kuroshimae* | *Heteropsammia cochlea* | 6 | 27 | 27 | 0 | 0 | 6 | - | 120 | 1 |
| ***Elysia* cf. *japonica*** | | | | | | | | | | | |
| **Trial Type** | **Prey Species** | **Predator Species** | **Prey Length (mm)** | **Mouth Length (mm)** | **Coral Diameter (mm)** | **Time on coral (mins)** | **Time at mouth (mins)** | **Ingestion Completed (mins)** | **Rejection Complete (mins)** | **P.I.O.L (mins)** | **Consumption Score** |
| in situ | *Elysia* cf. *japonica* | *Danafungia scruposa* | 7 | 28 | 120 | 0 | 0 | 1 | 26 | - | 0 |
| in situ | *Elysia* cf. *japonica* | *Fungia fungites* | 4 | 30 | 128 | 0 | 0 | 1 | - | 95 | 0 |
| in situ | *Elysia* cf. *japonica* | *Fungia fungites* | 8 | 25 | 160 | 0 | 0 | 1 | 6 | - | 0 |
| in situ | *Elysia* cf. *japonica* | *Danafungia scruposa* | 7 | 25 | 100 | 0 | 0 | 1 | - | 75 | 1 |
| in situ | *Elysia* cf. *japonica* | *Danafungia scruposa* | 7 | 60 | 200 | 0 | 0 | 4 | - | 74 | 0 |
| in situ | *Elysia* cf. *japonica* | *Pleuractis paumotensis* | 4 | 19 | 170 | 0 | 3 | 8 | - | 60 | - |
| in situ | *Elysia* cf. *japonica* | *Fungia fungites* | 11 | 36 | 180 | 0 | 0 | 2 | 17 | - | 0 |
| in situ | *Elysia* cf. *japonica* | *Fungia fungites* | 9 | 19 | 215 | 0 | 2 | 3 | 12 | - | 0 |
| in situ | *Elysia* cf. *japonica* | *Fungia fungites* | 8 | 43 | 330 | 0 | 18 | 20 | 26 | - | 0 |
| in situ | *Elysia* cf. *japonica* | *Danafungia scruposa* | 9 | 20 | 275 | 0 | 9 | 10 | 27 | - | 0 |
| ex situ | *Elysia* cf. *japonica* | *Danafungia scruposa* | 7 | 14 | 75 | 0 | 0 | 4 | - | 120 | 0.5 |
| ex situ | *Elysia* cf. *japonica* | *Fungia fungites* | 8 | 26 | 118 | 0 | 0 | 1 | - | 85 | 1 |
| ex situ | *Elysia* cf. *japonica* | *Danafungia scruposa* | 5 | 27 | 295 | 0 | 0 | 3 | - | 108 | 1 |
| ex situ | *Elysia* cf. *japonica* | *Danafungia scruposa* | 12 | 40 | 255 | 0 | 0 | 2 | - | 120 | 0.5 |
| ex situ | *Elysia* cf. *japonica* | *Pleuractis paumotensis* | 13 | 38 | 160 | 0 | 0 | 4 | 31 | - | 0.5 |
| ex situ | *Elysia* cf. *japonica* | *Danafungia scruposa* | 10 | 19 | 90 | 0 | 0 | 1 | 32 | - | 0 |
| ex situ | *Elysia* cf. *japonica* | *Danafungia scruposa* | 12 | 33 | 140 | 0 | 0 | 1 | 27 | - | 0 |
| ex situ | *Elysia* cf. *japonica* | *Pleuractis paumotensis* | 10 | 23 | 95 | 0 | 0 | 1 | 24 | - | 0.5 |
| ex situ | *Elysia* cf. *japonica* | *Pleuractis paumotensis* | 7 | 26 | 105 | 0 | 0 | 1 | - | 91 | - |
| ex situ | *Elysia* cf. *japonica* | *Pleuractis paumotensis* | 9 | 22 | 100 | 0 | 0 | 1 | - | 89 | - |
| in situ | *Elysia* cf. *japonica* | *Heteropsammia cochlea* | 11 | 24 | 38 | 0 | 0 | 2 | - | 90 | 0 |
| in situ | *Elysia* cf. *japonica* | *Heteropsammia cochlea* | 7 | 22 | 23 | 0 | 0 | 1 | - | 90 | 0 |
| in situ | *Elysia* cf. *japonica* | *Heteropsammia cochlea* | 14 | 24 | 24 | 0 | 0 | 1 | 22 | - | 0 |
| in situ | *Elysia* cf. *japonica* | *Heteropsammia cochlea* | 9 | 28 | 30 | 0 | 0 | 3 | 34 | - | 0 |
| in situ | *Elysia* cf. *japonica* | *Heteropsammia cochlea* | 8 | 22 | 21 | 0 | 0 | 6 | 3 | - | 0 |
| in situ | *Elysia* cf. *japonica* | *Heteropsammia cochlea* | 10 | 22 | 30 | 0 | 0 | 2 | - | - | 0 |
| in situ | *Elysia* cf. *japonica* | *Heteropsammia cochlea* | 9 | 17 | 23 | 0 | 0 | 9 | 8 | 50 | 0 |
| in situ | *Elysia* cf. *japonica* | *Heteropsammia cochlea* | 8 | 20 | 22 | 0 | 0 | 1 | - | 53 | 0 |
| in situ | *Elysia* cf. *japonica* | *Heteropsammia cochlea* | 6 | 16 | 18 | 0 | 0 | 2 | 1 | - | 0 |
| in situ | *Elysia* cf. *japonica* | *Heteropsammia cochlea* | 10 | 18 | 22 | 0 | 0 | 1 | - | 39 | 0 |
| ex situ | *Elysia* cf. *japonica* | *Heteropsammia cochlea* | 7 | 19 | 20 | 0 | 0 | 1 | 50 | - | 0 |
| ex situ | *Elysia* cf. *japonica* | *Heteropsammia cochlea* | 6 | 18 | 24 | 0 | 0 | 4 | 24 | - | 0 |
| ex situ | *Elysia* cf. *japonica* | *Heteropsammia cochlea* | 5 | 17 | 19 | 0 | 0 | 8 | 19 | - | 0 |
| ex situ | *Elysia* cf. *japonica* | *Heteropsammia cochlea* | 4 | 26 | 28 | 0 | 0 | 1 | - | 108 | 0 |
| ex situ | *Elysia* cf. *japonica* | *Heteropsammia cochlea* | 7 | 26 | 33 | 0 | 0 | 1 | 12 | - | 0 |
| ex situ | *Elysia* cf. *japonica* | *Heteropsammia cochlea* | 9 | 24 | 27 | 0 | 0 | 1 | - | 95 | 0 |
| ex situ | *Elysia* cf. *japonica* | *Heteropsammia cochlea* | 10 | 23 | 26 | 0 | 0 | 3 | 7 | - | 0 |
| ex situ | *Elysia* cf. *japonica* | *Heteropsammia cochlea* | 7 | 23 | 26 | 0 | 0 | 12 | 32 | - | 0 |
| ex situ | *Elysia* cf. *japonica* | *Heteropsammia cochlea* | 5 | 26 | 33 | 0 | 0 | 1 | - | 180 | 0 |
| ex situ | *Elysia* cf. *japonica* | *Heteropsammia cochlea* | 6 | 26 | 28 | 0 | 0 | 2 | - | 180 | 0.5 |
| ***Plakobranchus* cf. *ocellatus*** | | | | | | | | | | | |
| **Trial Type** | **Prey Species** | **Predator Species** | **Prey Length (mm)** | **Mouth Length (mm)** | **Coral Diameter (mm)** | **Time on coral (mins)** | **Time at mouth (mins)** | **Ingestion Completed (mins)** | **Rejection Complete (mins)** | **P.I.O.L (mins)** | **Consumption Score** |
| in situ | *Plakobranchus* cf. *ocellatus* | *Pleuractis paumotensis* | 33 | 25 | 195 | 0 | 11 | 16 | 26 | - | 0 |
| in situ | *Plakobranchus* cf. *ocellatus* | *Fungia fungites* | 23 | 32 | 265 | 0 | 26 | 33 | - | 33 | 0.5 |
| in situ | *Plakobranchus* cf. *ocellatus* | *Danafungia scruposa* | 11 | 32 | 225 | 0 | 1 | 12 | 24 | - | 0 |
| in situ | *Plakobranchus* cf. *ocellatus* | *Pleuractis paumotensis* | 9 | 21 | 195 | 0 | 16 | 18 | 26 | - | 0 |
| in situ | *Plakobranchus* cf. *ocellatus* | *Danafungia scruposa* | 13 | 13 | 200 | 0 | 7 | 20 | 33 | - | 0 |
| in situ | *Plakobranchus* cf. *ocellatus* | *Fungia fungites* | 35 | 19 | 270 | 0 | 5 | 15 | 35 | - | 0 |
| in situ | *Plakobranchus* cf. *ocellatus* | *Pleuractis paumotensis* | 40 | 32 | 220 | 0 | 3 | Incomplete Ingestion | 32 | - | 0 |
| in situ | *Plakobranchus* cf. *ocellatus* | *Danafungia scruposa* | 45 | 33 | 220 | 0 | 10 | Incomplete Ingestion | 49 | - | 0 |
| in situ | *Plakobranchus* cf. *ocellatus* | *Danafungia scruposa* | 42 | 35 | 250 | 0 | 22 | 42 | 51 | - | 0 |
| in situ | *Plakobranchus* cf. *ocellatus* | *Pleuractis paumotensis* | 40 | 40 | 180 | 0 | 4 | Incomplete Ingestion | 35 | - | 0 |
| ex situ | *Plakobranchus* cf. *ocellatus* | *Pleuractis paumotensis* | 8 | 21 | 185 | 0 | 6 | 13 | 23 | - | 0 |
| ex situ | *Plakobranchus* cf. *ocellatus* | *Danafungia scruposa* | 9 | 15 | 205 | 0 | 14 | 15 | 21 | - | 0 |
| ex situ | *Plakobranchus* cf. *ocellatus* | *Danafungia scruposa* | 38 | 42 | 355 | 0 | 5 | Incomplete Ingestion | 36 | - | 0 |
| ex situ | *Plakobranchus* cf. *ocellatus* | *Fungia fungites* | 40 | 33 | 230 | 0 | 7 | 25 | 34 | - | 0 |
| ex situ | *Plakobranchus* cf. *ocellatus* | *Danafungia scruposa* | 40 | 28 | 250 | 0 | 7 | Incomplete Ingestion | 37 | - | 0 |
| ex situ | *Plakobranchus* cf. *ocellatus* | *Fungia fungites* | 24 | 20 | 120 | 0 | 0 | 14 | 31 | - | 0 |
| ex situ | *Plakobranchus* cf. *ocellatus* | *Fungia fungites* | 30 | 21 | 110 | 0 | 0 | 18 | 38 | - | 0 |
| ex situ | *Plakobranchus* cf. *ocellatus* | *Fungia fungites* | 24 | 19 | 122 | 0 | 0 | 9 | 13 | - | 0 |
| ex situ | *Plakobranchus* cf. *ocellatus* | *Fungia fungites* | 27 | 25 | 140 | 0 | 0 | Incomplete Ingestion | 20 | - | 0 |
| ex situ | *Plakobranchus* cf. *ocellatus* | *Pleuractis paumotensis* | 10 | 30 | 220 | 0 | 6 | 13 | - | 90 | 0.5 |
| in situ | *Plakobranchus* cf. *ocellatus* | *Heteropsammia cochlea* | 32 | 30 | 34 | 0 | 0 | Incomplete Ingestion | - | - | 0 |
| in situ | *Plakobranchus* cf. *ocellatus* | *Heteropsammia cochlea* | 29 | 27 | 28 | 0 | 0 | Incomplete Ingestion | - | - | 0 |
| in situ | *Plakobranchus* cf. *ocellatus* | *Heteropsammia cochlea* | 16 | 24 | 28 | 0 | 0 | 16 | 25 | - | 0 |
| in situ | *Plakobranchus* cf. *ocellatus* | *Heteropsammia cochlea* | 28 | 21 | 24 | 0 | 0 | Incomplete Ingestion | - | - | 0 |
| in situ | *Plakobranchus* cf. *ocellatus* | *Heteropsammia cochlea* | 17 | 18 | 21 | 0 | 0 | 12 | 14 | - | 0 |
| in situ | *Plakobranchus* cf. *ocellatus* | *Heteropsammia cochlea* | 21 | 25 | 28 | 0 | 0 | Incomplete Ingestion | - | - | 0 |
| in situ | *Plakobranchus* cf. *ocellatus* | *Heteropsammia cochlea* | 25 | 22 | 22 | 0 | 0 | Incomplete Ingestion | - | - | 0 |
| in situ | *Plakobranchus* cf. *ocellatus* | *Heteropsammia cochlea* | 20 | 29 | 32 | 0 | 0 | 2 | 8 | - | 0 |
| in situ | *Plakobranchus* cf. *ocellatus* | *Heteropsammia cochlea* | 20 | 26 | 22 | 0 | 0 | Incomplete Ingestion | - | - | 0 |
| in situ | *Plakobranchus* cf. *ocellatus* | *Heteropsammia cochlea* | 26 | 23 | 26 | 0 | 0 | Incomplete Ingestion | - | - | 0 |
| ex situ | *Plakobranchus* cf. *ocellatus* | *Heteropsammia cochlea* | 26 | 28 | 28 | 0 | 0 | Incomplete Ingestion | - | - | 0 |
| ex situ | *Plakobranchus* cf. *ocellatus* | *Heteropsammia cochlea* | 22 | 28 | 27 | 0 | 0 | Incomplete Ingestion | - | - | 0 |
| ex situ | *Plakobranchus* cf. *ocellatus* | *Heteropsammia cochlea* | 22 | 28 | 31 | 0 | 0 | Incomplete Ingestion | - | - | 0 |
| ex situ | *Plakobranchus* cf. *ocellatus* | *Heteropsammia cochlea* | 19 | 26 | 24 | 0 | 0 | Incomplete Ingestion | - | - | 0 |
| ex situ | *Plakobranchus* cf. *ocellatus* | *Heteropsammia cochlea* | 20 | 29 | 32 | 0 | 0 | Incomplete Ingestion | - | - | 0 |
| ex situ | *Plakobranchus* cf. *ocellatus* | *Heteropsammia cochlea* | 12 | 24 | 25 | 0 | 0 | 14 | 19 | - | 0 |
| ex situ | *Plakobranchus* cf. *ocellatus* | *Heteropsammia cochlea* | 25 | 24 | 24 | 0 | 0 | Incomplete Ingestion | - | - | 0 |
| ex situ | *Plakobranchus* cf. *ocellatus* | *Heteropsammia cochlea* | 15 | 29 | 29 | 0 | 0 | Incomplete Ingestion | - | - | 0 |
| ex situ | *Plakobranchus* cf. *ocellatus* | *Heteropsammia cochlea* | 20 | 30 | 28 | 0 | 0 | Incomplete Ingestion | - | - | 0 |
| ex situ | *Plakobranchus* cf. *ocellatus* | *Heteropsammia cochlea* | 26 | 29 | 29 | 0 | 0 | Incomplete Ingestion | - | - | 0 |
