# Supplementary material for: Selective consumption of sacoglossan sea slugs (Mollusca: Gastropoda) by scleractinian corals (Cnidaria: Anthozoa)
Source: PLoS One. 2019 Apr 29;14(4):e0215063. doi: 10.1371/journal.pone.0215063 (PMC6488191; doi:10.1371/journal.pone.0215063)
Supplement: S2 Table — (DOCX) [file pone.0215063.s002.docx]

|  | **In-situ** | | | | **Ex-situ** | | | |
| --- | --- | --- | --- | --- | --- | --- | --- | --- |
| **Species** | *Danafungia scruposa* | *Fungia fungites* | *Pleuractis paumotensis* | *Heteropsammia cochlea* | *Danafungia scruposa* | *Fungia fungites* | *Pleuractis paumotensis* | *Heteropsammia cochlea* |
| *Costasiella* cf. *kuroshimae* | 2.25 ± 0.68 (n=4) | 9  (n=1) | 3.2 ± 0.58 (n=5) | 4.6 ± 1.82 (n=10) | 3 ± 1.22  (n=4) | 5.83 ± 3.49 (n=6) | N/A | 4.4 ± 0.81 (n=10) |
| *Costasiella usagi* | 4.5 ± 2.5  (n=2) | 2.4 ± 0.75 (n=5) | 4.33 ± 1.67 (n=3) | 2.3 ± 0.37 (n=10) | 3.43 ± 0.72 (n=7) | 4.33 ± 1.45 (n=3) | N/A | 2.7 ± 0.67 (n=10) |
| *Elysia* cf. *japonica* | 4 ± 2.12  (n=4) | 5.4 ± 3.67 (n=5) | 8  (n=1) | 2.8 ± 0.84 (n=10) | 2.2 ± 0.58 (n=5) | 1  (n=1) | 1.75 ± 0.75 (n=4) | 3.4 ± 1.19 (n=10) |
| *Elysia pusilla* | 5  (n=1) | 9.25± 3.75 (n=4) | 5 ± 0.89  (n=5) | 8.5 ± 2.45 (n=10) | 1.33 ± 0.33 (n=3) | 7.83 ± 2.82 (n=6) | 2 (n=1) | 7.9 ± 2.62 (n=10) |
| *Plakobranchus* cf. *ocellatus* | 24.67 ± 8.85 (n=4) | 24 ± 9  (n=2) | 17 ± 4.92  (n=4) | 10 ± 1.87 (n=10) | 15 ± 5  (n=3) | 16 ± 3.77  (n=5) | 13 ± 0  (n=2) | 14 ± 1.4  (n=10) |
| *Plakobranchus* cf. *papua* | 7.5 ± 2.65 (n=3) | 4.5 ± 0.5  (n=2) | 8.2 ± 1.69 (n=5) | 16 ± 1.6  (n=10) | N/A | N/A | 5.38 ± 1.24 (n=10) | 7 ± 0.7  (n=10) |
