# Supplementary material for: Selective consumption of sacoglossan sea slugs (Mollusca: Gastropoda) by scleractinian corals (Cnidaria: Anthozoa)
Source: PLoS One. 2019 Apr 29;14(4):e0215063. doi: 10.1371/journal.pone.0215063 (PMC6488191; doi:10.1371/journal.pone.0215063)
Supplement: S3 Table — (DOCX) [file pone.0215063.s003.docx]

|  | **In-situ** | | | | **Ex-situ** | | | |
| --- | --- | --- | --- | --- | --- | --- | --- | --- |
| **Species** | *Danafungia scruposa* | *Fungia fungites* | *Pleuractis paumotensis* | *Heteropsammia cochlea* | *Danafungia scruposa* | *Fungia fungites* | *Pleuractis paumotensis* | *Heteropsammia cochlea* |
| *Costasiella* cf. *kuroshimae* | N/A  (n=4) | N/A  (n=1) | 4 ± 0.8  (n=5) | N/A  (n=10) | N/A  (n=4) | 17 ± 2.83  (n=6) | N/A | 14.67 ± 2.30 (n=10) |
| *Costasiella usagi* | N/A  (n=2) | 18 ± 3.6  (n=5) | N/A  (n=3) | 9 ± 0.9  (n=10) | N/A  (n=7) | 15 ± 7.21  (n=3) | N/A | 41 ± 7.96 (n=10) |
| *Elysia* cf. *japonica* | 26.5 ± 0.35 (n=4) | 15.25 ± 4.48 (n=5) | N/A  (n=1) | 13.6 ± 3.73 (n=10) | 29.5 ± 7.27 (n=5) | N/A  (n=1) | 27.5 ± 8.07 (n=4) | 24 ± 5.35 (n=10) |
| *Elysia pusilla* | N/A  (n=1) | N/A  (n=4) | N/A  (n=5) | N/A  (n=10) | N/A  (n=3) | N/A  (n=6) | N/A  (n=1) | 18 ± 1.8  (n=10) |
| *Plakobranchus* cf. *ocellatus* | 39.25 ± 6.49 (n=4) | 35 ± 17.5  (n=2) | 29.75± 2.25 (n=4) | 15.67 ± 2.72 (n=10) | 31.33 ± 5.17 (n=3) | 25.83 ± 4.41 (n=5) | 23 ± 11.5  (n=2) | 19 ± 1.9  (n=10) |
| *P.* cf. *papua* | 12.33 ± 2.60 (n=3) | 16.5 ± 3.5 (n=2) | 22 ± 4.82  (n=5) | 9.57 ± 2.13 (n=10) | N/A | N/A | 13.71 ± 2.42 (n=10) | 14.5 ± 2.16 (n=10) |
