# Supplementary material for: Selective consumption of sacoglossan sea slugs (Mollusca: Gastropoda) by scleractinian corals (Cnidaria: Anthozoa)
Source: PLoS One. 2019 Apr 29;14(4):e0215063. doi: 10.1371/journal.pone.0215063 (PMC6488191; doi:10.1371/journal.pone.0215063)
Supplement: S4 Table — (DOCX) [file pone.0215063.s004.docx]

|  | **In-situ** | | | | **Ex-situ** | | | |
| --- | --- | --- | --- | --- | --- | --- | --- | --- |
| **Species** | *Danafungia scruposa* | *Fungia fungites* | *Pleuractis paumotensis* | *Heteropsammia cochlea* | *Danafungia scruposa* | *Fungia fungites* | *Pleuractis paumotensis* | *Heteropsammia cochlea* |
| *Costasiella* cf. *kuroshimae* | 0.5± 0.18  (n=4) | N/A  (n=1) | 0.5 ± 0.2  (n=5) | 0.4 ± 0.1 (n=10) | 1 ± 0  (n=4) | 0.33 ± 0.17 (n=6) | N/A | 0.3 ± 0.11 (n=10) |
| *Costasiella usagi* | 0.75 ± 0.25 (n=2) | 0.2± 0.12  (n=5) | 0.5 ± 0.17 (n=3) | 0.35 ± 0.08 (n=10) | 0.83 ± 0.18 (n=7) | 0 ± 0  (n=3) | N/A | 0.5 ± 0.13 (n=10) |
| *Elysia* cf. *japonica* | 0.25 ± 0.25 (n=4) | 0 ± 0  (n=5) | N/A  (n=1) | 0 ± 0  (n=10) | 0.4 ± 0.19 (n=5) | 1  (n=1) | 0.5 ± 0.14 (n=4) | 0.05 ± 0.05 (n=10) |
| *Elysia pusilla* | N/A  (n=1) | 1 ± 0  (n=4) | 0.5 ± 0.12 (n=5) | 0.6 ± 0.1 (n=10) | 1 ± 0.33  (n=3) | 0.5 ± 0.08 (n=6) | NA  (n=1) | 0.69 ± 0.14 (n=10) |
| *Plakobranchus* cf. *ocellatus* | 0 ± 0  (n=4) | 0.25 ± 0.25 (n=2) | 0 ± 0  (n=4) | 0 ± 0  (n=10) | 0 ± 0  (n=3) | 0 ± 0  (n=5) | 0.25 ± 0.25 (n=2) | 0 ± 0  (n=10) |
| *P.* cf. *papua* | 0 ± 0  (n=3) | 0 ± 0  (n=2) | 0 ± 0  (n=5) | 0 ± 0  (n=10) | N/A | N/A | 0 ± 0  (n=10) | 0 ± 0  (n=10) |
