# Supplementary material for: Selective consumption of sacoglossan sea slugs (Mollusca: Gastropoda) by scleractinian corals (Cnidaria: Anthozoa)
Source: PLoS One. 2019 Apr 29;14(4):e0215063. doi: 10.1371/journal.pone.0215063 (PMC6488191; doi:10.1371/journal.pone.0215063)
Supplement: S5 Table — (DOCX) [file pone.0215063.s005.docx]

|  | **In-situ** | | | **Ex-situ** | | |
| --- | --- | --- | --- | --- | --- | --- |
| **Species** | *Danafungia scruposa* | *Fungia fungites* | *Pleuractis paumotensis* | *Danafungia scruposa* | *Fungia fungites* | *Pleuractis paumotensis* |
| *Costasiella* cf. *kuroshimae* | 25  (n=1) | 20.63  (n=1) | 28.97 ± 16.55 (n=3) | 7.49 ± 3.88 (n=2) | N/A | N/A |
| *Costasiella usagi* | 6.08  (n=1) | 13.75  (n=1) | 17.82 ± 8.08 (n=2) | 22.67  (n=1) | 22.33  (n=1) | N/A |
| *Elysia* cf. *japonica* | 14.17  (n=1) | 12.36 ± 3.97 (n=2) | 8.39  (n=1) | N/A | N/A | N/A |
| *Elysia pusilla* | 7.13  (n=1) | 15  (n=1) | 14.86 ± 2.99 (n=3) | N/A | 10.25  (n=1) | N/A |
| *Plakobranchus* cf. *ocellatus* | 6.97 ± 1.30 (n=3) | 6.42 ± 1.94 (n=2) | 8.67 ± 2.39 (n=4) | 11.79 ± 3.86 (n=2) | 14.07  (n=1) | 6.24 ± 1.68 (n=2) |
